# Insights into the Adsorption of Carbon Dioxide in Zeolites ITQ-29 and 5A Based on Kinetic Measurements and Molecular Simulations

**DOI:** 10.3390/nano15141077

**Published:** 2025-07-11

**Authors:** Magdy Abdelghany Elsayed, Shixue Zhou, Xiaohui Zhao, Gumawa Windu Manggada, Zhongyuan Chen, Fang Wang, Zhijuan Tang

**Affiliations:** 1 College of Energy and Mining Engineering, Shandong University of Science and Technology, Qingdao 266590, China; magdyabdelghany10@163.com (M.A.E.); 18535824890@163.com (X.Z.); gumawawindu@gmail.com (G.W.M.); 13156213199@163.com (F.W.); 18791101514@163.com (Z.T.); 2 Department of Mining and Petroleum Engineering, Faculty of Engineering, Al-Azhar University, Cairo 11884, Egypt; 3 College of Chemical and Biological Engineering, Shandong University of Science and Technology, Qingdao 266590, China; chenzhongyuan299@163.com

**Keywords:** adsorption kinetics, LTA zeolite, molecular simulation, isosteric heat, adsorption energy

## Abstract

Understanding the adsorption mechanism is essential for developing efficient technologies to capture carbon dioxide from industrial flue gases. In this work, laboratory measurements, density functional theory calculations, and molecular dynamics simulations were employed to study CO_2_ adsorption and diffusion behavior in LTA-type zeolites. The CO_2_ adsorption isotherms measured in zeolite 5A are best described by the Toth model. Thermodynamic analysis indicates that the adsorption process is spontaneous and exothermic, with an enthalpy change of −44.04 kJ/mol, an entropy change of −115.23 J/(mol·K), and Gibbs free energy values ranging from −9.68 to −1.03 kJ/mol over the temperature range of 298–373 K. The isosteric heat of CO_2_ adsorption decreases from 40.35 to 21.75 kJ/mol with increasing coverage, reflecting heterogeneous interactions at Ca^2+^ and Na^+^ sites. The adsorption kinetics follow a pseudo-first-order model, with an activation energy of 2.24 kJ/mol, confirming a physisorption mechanism. The intraparticle diffusion model indicates that internal diffusion is the rate-limiting step, supported by a significant reduction in the diffusion rate. The DFT calculations demonstrated that CO_2_ exhibited a −35 kJ/mol more negative adsorption energy in zeolite 5A than in zeolite ITQ-29, attributable to strong interactions with Ca^2+^/Na^+^ cations in 5A that were absent in the pure silica ITQ-29 framework. The molecular dynamics simulations based on molecular force fields indicate that CO_2_ diffuses more rapidly in ITQ-29, with a diffusion coefficient measuring 2.54 × 10^−9^ m^2^/s at 298 K, whereas it was 1.02 × 10^−9^ m^2^/s in zeolite 5A under identical conditions. The activation energy for molecular diffusion reaches 5.54 kJ/mol in zeolite 5A, exceeding the 4.12 kJ/mol value in ITQ-29 by 33%, which accounts for the slower diffusion kinetics in zeolite 5A. There is good agreement between experimental measurements and molecular simulation results for zeolite 5A across the studied temperature and pressure ranges. This confirms the accuracy and reliability of the selected simulation parameters and allows for the study of zeolite ITQ under similar simulation conditions. This research provides insights into CO_2_ adsorption energetics and diffusion within LTA-type zeolite frameworks, supporting the rational design of high-performance adsorbents for industrial gas separation.

## 1. Introduction

Carbon dioxide (CO_2_), a potent greenhouse gas, is a primary driver of global warming, resulting in climate change due to its heat-trapping effects in the atmosphere [1,2]. Industrial CO_2_ capture methods such as absorption, membrane separation, and cryogenic distillation are widely used [3,4]. However, these processes often require high energy input and face challenges related to selectivity and efficiency [5]. In contrast, the adsorption process using porous materials offers advantages including lower energy consumption, high efficiency, and operational simplicity [6,7]. As a result, for CO_2_, this has attracted attention [8], supported by the development of diverse adsorbents, including activated carbon (AC) [9,10], metal organic frameworks (MOFs) [11,12], covalent organic frameworks (COFs) [13,14], and zeolites [15,16].

Zeolites are widely used for adsorptive separation due to their pore-type structures, thermal stability, cost-effectiveness, and high surface areas [17,18], and the adsorption performance of zeolites can be tuned by adjusting pore size, framework composition, and extraframework cations [19]. The LTA-type zeolites have highly ordered three-dimensional microporous frameworks, with α-cagesconnected through eight-membered ring windows with an aperture of approximately 4.1 Å, where the α-cage, approximately 11.4 Å in diameter, is symmetrically surrounded by eight β-cages, which are arranged in a cubic lattice and connected through double four-ring units. The interconnected channels and suitable pore sizes allow small guest molecules to diffuse freely throughout the structure, providing isotropic diffusion pathways [20,21,22,23]. Among the LTA-type zeolites, zeolite 5A is employed in various adsorption-based separation processes, such as distinguishing between branched and normal paraffins, as well as in the separation of nitrogen and oxygen from air [24]. The high affinity of zeolite 5A for CO_2_ is attributed to the strong electrostatic interactions between CO_2_ and the cations located within the zeolite structure [25].

Previous studies have investigated CO_2_ adsorption in zeolite 5A; however, key aspects, such as the detailed molecular adsorption mechanisms and the effects of framework composition and cation distribution, require further extensive investigation. Boonchuay et al. [26] reported that CO_2_ adsorption is a diffusion-controlled physical process, influenced by CO_2_ concentration, temperature, and feed flow rate. Saha et al. [27] demonstrated that zeolite 5A, compared with MOF-5 and MOF-177, exhibited superior selectivity and efficiency in the removal of CO_2_ and N_2_O from air, as well as in CO_2_/CH_4_ separation. Khoramzadeh et al. [28] mentioned that zeolite 5A, especially at lower pressures, exhibits higher CO_2_ adsorption capacity and selectivity in CO_2_/N_2_ separation.

A comprehensive understanding of isotherms, thermodynamics, and kinetics is essential for optimizing CO_2_ capture systems. Isotherm studies elucidate the relationship between CO_2_ concentration and adsorption capacity, providing insight into adsorption mechanisms [29]. Thermodynamic parameters, including entropy change and heat of adsorption, are analyzed to assess the spontaneity and feasibility of adsorption processes [30]. Kinetics analysis is used to determine the rate at which CO_2_ molecules are captured by adsorbents, which directly includes the efficiency of the process and the time required to reach equilibrium, thereby impacting the overall performance and scalability of CO_2_ capture systems [31]. Kinetic models have various mathematical formulations, like pseudo-first-order and pseudo-second-order models, which are important for describing the diffusion mechanism. The kinetic models were used to depict the rate of adsorption or desorption processes onto a solid surface, assisting in the optimization and design of CO_2_ capture systems by determining the required operational conditions to maximize efficiency and minimize energy consumption, in which the operations include temperature, flow rates, and residence time [32]. Diverse kinetic models have been applied to explore CO_2_ adsorption on different adsorbents, offering a deeper understanding of the main adsorption mechanisms [33,34]. A comprehensive analysis of these factors can help optimize operational conditions, select suitable adsorbents, and enhance CO_2_ capture efficiency for carbon capture applications.

Additionally, computational modeling has become a valuable tool for studying adsorption mechanisms. Classical simulation methods like Grand Canonical Monte Carlo (GCMC) and molecular dynamics (MD) simulations, along with quantum mechanical approaches, like density functional theory (DFT) calculation, are used to predict CO_2_ binding sites, cation interactions, and diffusion pathways within zeolite frameworks [35,36]. Wang et al. [37] studied the adsorption of CO_2_, H_2_O, SO_2_, N_2_, O_2_, NO, and NO_2_ in zeolites 4A (LTA Framework), MFI, and MOR using Monte Carlo and MD simulations at temperatures ranging from 253–333 K. Among them, zeolite 4A showed the highest CO_2_ capacity and the best equilibrium-based separation performance for CO_2_. Golchoobi et al. [38] applied GCMC simulations to study CO_2_ and CH_4_ adsorption in zeolite 4A, using a universal force field with van der Waals (vdW) interactions modeled via the atom-based method and electrostatic contributions calculated using Ewald summation, which well matched experimental data results, revealing a maximum CO_2_ uptake of 3.17 mol/kg. Similarly, Akten et al. [39] used GCMC to examine CO_2_, N_2_, and H_2_ adsorption in dehydrated Na-A, calibrating interatomic potentials against single-component isotherms. The simulations confirmed strong CO_2_ selectivity over H_2_ and N_2_, though selectivity diminished at elevated pressures. In addition, Wang et al. [40] employed validated molecular simulations to investigate the adsorption thermodynamics and kinetics of CO_2_ and six combustion gases on commercial zeolites 13X and 5A with CO_2_ uptake reduced by 80% in zeolite 13X and 83% in zeolite 5A due to competitive adsorption with H_2_O. Okello et al. [41] employed molecular simulations to evaluate CO_2_ adsorption on zeolites, revealing that Linde type A zeolite exhibited the highest uptake capacity of 69.88% at 298 K and 1000 kPa, with the results closely matching experimental data. Li et al. [42] conducted DFT calculations to assess CO_2_ adsorption in metal-exchanged zeolites (Y, CHA, ZSM-5, and A). Their findings show that monovalent cations follow the trend Li^+^ > Na^+^ > K^+^ > Cu^+^ in adsorption strength, while divalent cations (Mg^2+^, Ca^2+^) in zeolite Y exhibit particularly high affinity. Integrating experimental and computational approaches to estimate the CO_2_ adsorption in zeolites enhances the understanding and optimization of the adsorption process [43]. Laboratory experiments, such as adsorption isotherms, provide empirical insights into CO_2_ capture by zeolites under various conditions. Computational modeling, like molecular simulations or DFT calculations, predicts the molecular-level interactions within the zeolite frameworks.

In this work, experimental measurements and molecular simulations were combined to investigate CO_2_ adsorption behavior in two LTA-type zeolites, 5A and ITQ-29. Specifically, our study aims to (i) elucidate the molecular-level adsorption mechanisms of CO_2_ within the LTA framework under varying conditions, (ii) evaluate the influence of framework composition and cation distribution on CO_2_ sorption performance, and (iii) provide comparative insights into adsorption energetics and diffusion dynamics that complement and extend the existing literature. Experimentally, temperature-dependent adsorption isotherms (298–373 K) and kinetics were measured in zeolite 5A, with equilibrium data fitted using Langmuir, Freundlich, Sips, and Toth models. Kinetic behavior was evaluated through pseudo-first-order, pseudo-second-order, and intraparticle diffusion models. The simulation study was applied to investigate CO_2_ adsorption in two LTA-type zeolites, ITQ-29 and 5A, using first-principle calculations based on density functional theory calculations and classical molecular simulation methods with a molecular force field. Key properties such as atomic charges, adsorbate configurations, and adsorption energies were evaluated. Additionally, adsorption isotherms and molecular diffusion dynamics were simulated to gain deeper insight into the interactions between CO_2_ and zeolite frameworks.

## 2. Materials and Methods

### 2.1. Experimental Setup and Procedure

Pristine zeolite 5A (LTA type), Ca*_n_*Na_12-2_*_n_*[(AlO_2_)_12_(SiO_2_)_12_]·xH_2_O, in powder form (<10 μm, Sigma-Aldrich, St. Louis, MO, USA, catalog number 233676), which has a fixed Ca^2+^/Na^+^ molar ratio of ~0.96, as determined in this study based on elemental composition data obtained from energy-dispersive-X-ray spectroscopy (EDS) spectra, and high-purity CO_2_ (≥99.999%, Qingdao Deyi Gas Co., Ltd., Qingdao, China) were used. X-ray diffraction (XRD) was conducted using an Ultima IV multi-functional diffractometer (Rigaku, Tokyo, Japan) with Cu Kα radiation in the 2*θ* range of 5–50° at a scanning speed of 2°/min to confirm the crystalline structure and phase purity of the zeolite. Fourier transform infrared spectroscopy (FTIR) analysis was performed on a Nicolet™ iS™ 50 FTIR spectrometer (Thermo Fisher Scientific, Waltham, MA, USA) within the 4000–400 cm^−1^ spectral range. The sample was prepared using the KBr pellet technique to identify functional groups and analyze the structural features of the zeolite framework. Nitrogen adsorption–desorption measurements were performed on a Micromeritics 3-Flex (Micromeritics Instrument Corporation, Norcross, GA, USA) at liquid nitrogen temperature to determine the specific surface area, pore size distribution, and porosity characteristics of the zeolite, analyzed using the Brunauer–Emmett–Teller (BET) method and the nonlocal density functional theory (NLDFT) method. The morphology and elemental composition of the zeolite were examined using a Tescan TS 5130MM scanning electron microscope (SEM) equipped with an EDS detector (Oxford Instruments, Abingdon, UK, active crystal area-50 mm^2^) to investigate the surfacetexture, particle size, and elemental distribution. Adsorption experiments were conducted using an automatic Sievert-type apparatus (PCTPro-2000, Setaram, Caluire-et-Cuire, France) to evaluate adsorption isotherms, with a purification process performed prior to testing by heating the sample at 523 K for 2 h under vacuum to remove impurities such as water and strongly adsorbed contaminants within the zeolite pores, followed by adsorption measurements at temperatures of 298, 323, 348, and 373 K and pressures up to 200 kPa, while adsorption kinetics experiments were conducted at 100, 200, and 300 kPa.

### 2.2. Adsorption Isotherm Models and Isosteric Heat

The isotherm models, including Langmuir, Freundlich, Sips, and Toth, were applied to analyze the equilibrium adsorption behavior of CO_2_ in zeolite 5A. The Langmuir model in Equation (1) assumes monolayer adsorption on a homogeneous surface with identical binding sites [44], whereas the Freundlich model in Equation (2) describes heterogeneous adsorption with varying site energies [45]. The Sips model, shown in Equation (3), combines the characteristics of the Freundlich and Langmuir models, accommodating both multilayer and monolayer adsorption [46]. The Toth model in Equation (4) further refines the Langmuir approach by integrating surface heterogeneity, providing a more comprehensive description of adsorption behavior [47]. The adsorption equilibrium parameters were determined by fitting experimental data with these models. It is necessary for understanding the thermodynamic characteristics exhibited during the adsorption process. The equations for these models are as follows:

(1) $$\frac{q}{q_{m}}\;=\;\frac{kp}{1+kp}$$

(2) $$q\;={\;\left(kp\right)}^{\frac{1}{n}}$$

(3) $${q\;=q}_{m}\frac{{\left(kp\right)}^{n}}{1+{\left(kp\right)}^{n}}$$

(4) $$q\;={\;q}_{m}\frac{kp}{{\left[1+{\left(kp\right)}^{m}\right]}^{\frac{1}{m}}}$$

where *k* represents the respective adsorption constants associated with the Langmuir, Freundlich, Sips, and Toth isotherm models; *p* is the applied pressure on the adsorbed molecules (kPa); *n* represents a parameter that describes the heterogeneity of the adsorption surface and the degree of favorability of the adsorption; *q*_m_ is the maximum adsorption uptake of CO_2_ (mmol/g); and *m* is the heterogeneity parameter for the Toth model [30]. The normalized standard deviation (Δ*Q*), based on the residuals between the experimental data and the values predicted by the adsorption models, was calculated using Equation (5) [48].

(5) $$\Delta Q\;=\;\;\sqrt{\frac{\sum_{i}^{n}{\left(q_{\mathrm{actual}}-\frac{q_{\mathrm{predicted}}}{q_{\mathrm{actual}}}\right)}^{2}}{n-1}}$$

where Δ*Q* is the normalized standard deviation; *n* is the total number of data points, *q*_actual_ is the experimentally measured value; and *q*_predicted_ is the value calculated by the adsorption model.

The separation factor (*R*_L_) is a crucial parameter to evaluate isotherms, representing the essential characteristics of adsorption behavior. It is calculated using Equation (6), where *k* is the isotherm model adsorption equilibrium constant and *C*_0_ is the initial concentration of the adsorbate. The *R*_L_ value helps to determine the favorability of adsorption; *R*_L_ < 1 indicates favorable adsorption, *R*_L_ = 1 indicates linear adsorption (no adsorption process is taking place), *R*_L_ > 1 indicates unfavorable adsorption, and *R*_L_ = 0 denotes irreversible adsorption [49].

(6) $$R_{L}=\;\frac{1}{1+kC_{0}}$$

The isosteric heat of adsorption (*Q*_st_) was estimated using the Clausius–Clapeyron relationship to the van’t Hoff (Equation (7)), which relates the natural logarithm of the equilibrium pressure (*p*) to the standard enthalpy of adsorption (∆*H*^0^) and entropy of adsorption (∆*S*^0^), where R is the universal gas constant, and *T* is the temperature.

(7) $$\;\ln(p)=\frac{\Delta S^{0}}{R}-\frac{\Delta H^{0}}{RT}$$

By differentiating ln(*p*) with respect to temperature (*T*), the isosteric heat of adsorption (*Q*_st_) was determined using Equation (8). This equation was derived from the Clausius–Clapeyron relationship and is commonly used to calculate the heat of adsorption from adsorption isotherms at different temperatures under constant coverage (Γ). The isosteric heat of adsorption refers to the energy required to desorb one mole of adsorbed molecules from the surface of the adsorbent at a constant temperature and provides valuable insights into the strength and nature of the adsorbate–adsorbent interactions, with higher values indicating stronger interactions and vice versa.

(8) $$Q_{\mathrm{st}}=\;{\left(\frac{\partial \ln\left(p\right)}{\partial T}\right)}_{\Gamma }RT^{2}$$

### 2.3. Modeling and Simulation Techniques

#### 2.3.1. Optimized Structural Modeling and Density Functional Theory Calculations

In this work, two LTA-type zeolites were used as adsorbents, zeolite ITQ-29 and zeolite 5A. The cell model of zeolite ITQ-29 was built using the atomic coordinates and lattice parameters reported in the literature [50], corresponding to *a* = *b* = *c* = 23.70 Å and a composition of Si_192_O_384_. The unit cell model of zeolite 5A was similarly constructed using atomic coordinates from the literature [51], with *a* = *b* = *c* = 24.84 Å and composition Ca_32_Na_32_Si_96_A_96_O_384_. These structures were applied in all calculations, with the atomic coordinates and lattice parameters obtained from experimental data reported in the cited literature.

Subsequent geometry optimization was performed using the DMol^3^ module in the Materials Studio 2017 software (Accelrys Software Inc., San Diego, CA, USA) based on DFT calculations. The Perdew–Burke–Ernzerhof (PBE) functional, a variant of the generalized gradient approximation (GGA), was implemented to evaluate nonlocal exchange-correlation energy [52]. The electronic wave functions were expanded using a double numerical plus polarization (DNP) basis set to ensure high precision [53], with the basis file version specified as 4.4. The self-consistent field (SCF) calculations were conducted with a stringent convergence tolerance of 10^−6^ Hartree (Ha). To enhance SCF convergence efficiency, the direct inversion in the iterative subspace (DIIS) method, preconditioner, and orbital smearing were activated, with respective parameters configured to 6, 4.0 *a*_0_^−1^ and 0.005 Ha. Hexadecapole approximation was selected for multipolar expansion to accurately model the electron density distribution. A global orbital cut-off scheme was applied, with a global cut-off radius of 4.8 Å, ensuring sufficient representation of the electronic interactions. For Brillouin zone sampling, only the gamma point was utilized, as the system size ensured that the electronic wave functions exhibited sufficient smoothness in real space, rendering additional k-point sampling unnecessary. Geometry optimization was executed with convergence thresholds for energy, maximum force, and maximum displacement set at 1.0 × 10^−5^ Ha, 0.002 Ha Å^−1^, and 0.005 Å, respectively.

Structural optimization was carried out using DFT calculations to obtain a highly accurate atomic structure that incorporates electronic effects and optimized cell parameters, which are not fully accounted for by classical force fields. The optimized structure was subsequently used in classical molecular simulations to ensure that adsorption properties were evaluated based on a realistic and energetically reliable configuration, as demonstrated in previous studies on CO_2_ adsorption in zeolites [54].

**Figure 1:**
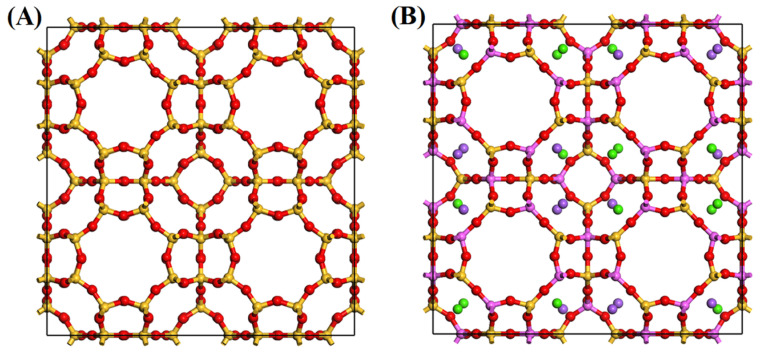
Structural models of zeolite (**A**) ITQ-29 and (**B**) zeolite 5A. Atomic species are yellow (Si), red (O), pink (Al), purple (Na), and green (Ca). Lattice parameters for zeolite ITQ-29 are *a* = *b* = *c* = 23.70 Å [50], and for zeolite 5A, *a* = *b* = *c* = 24.84 Å. Chemical formulas are Si_192_O_384_ for zeolite ITQ-29, and Ca_32_Na_32_Si_96_Al_96_O_384_ for zeolite 5A [51].

The optimized structures of ITQ-29 and zeolite 5A (Figure 1) display the characteristic LTA-type frameworks, composed of three fundamental building units: double four-membered rings (D4Rs), sodalite cages (β-cages), and supercages (α-cages). In this configuration, the β-cages form a simple cubic packing and are interconnected via D4R units, with each α-cage centrally positioned and surrounded by eight β-cages. In the case of zeolite 5A, Na^+^ and Ca^2+^ cations preferentially occupy positions near the 6-membered rings of the α-cages, as illustrated in Figure S1. Importantly, the DFT geometry optimization maintained both the atomic coordinates and unit cell parameters in close agreement with the established literature values [50,51] (see Tables S1 and S2), thereby validating the reliability of the computational approach while preserving the intrinsic structural topology of the LTA framework.

The X-ray diffraction patterns of the cell models, simulated using the Powder Diffraction task in the Reflex module of Materials Studio software, are presented in Figure S2A,B for zeolite ITQ-29 and zeolite 5A, respectively. A complete description of the simulation procedure is provided in the Supplementary Materials.

Hirshfeld population analysis was used to determine the atomic charges in the zeolite frameworks, while Mulliken charge analysis was applied to the CO_2_ molecule in a vacuum. The average atomic charges in zeolites ITQ-29 and 5A are listed in Table S3 and are consistent with the literature values [55]. When these charges were used for adsorption simulations, the resulting simulated adsorption capacities aligned with experimental data more closely than those obtained using COMPASS, ESP, QEq, or Gasteiger charges, as shown in Figures S3 and S4.

The DFT calculations were used to optimize the geometries of both zeolite ITQ-29 and cation-exchanged zeolite 5A cells with CO_2_ molecules. In the zeolite ITQ-29 model, a CO_2_ molecule was placed near an Si atom in a 6-membered ring, with the distance between the Si atom and one of the oxygen atoms of CO_2_ being shorter than the sum of their atomic radius for both atoms. During the optimization, only the atoms shown as balls in Figure S5 were allowed to relax, while the atoms represented as sticks were kept fixed.

In addition, a CO_2_ molecule was introduced near the Ca^2+^ cation in various configurations for zeolite 5A, as shown in Figure S5, which also includes zeolite ITQ-29. Configuration (A) corresponds to zeolite ITQ-29, while the configurations represent zeolite 5A with the initial CO_2_ placements labeled as (B), (C), (D), and (E), respectively. The adsorption energy of CO_2_ was calculated by subtracting the energies of the zeolite and CO_2_ molecule in their states from the total energy of the optimized zeolite/CO_2_ system, allowing for an accurate assessment of the interaction energy between CO_2_ and the zeolite framework.

#### 2.3.2. Grand Canonical Monte Carlo Simulations

The GCMC method, combined with periodic boundary conditions, was employed to simulate CO_2_ adsorption using the adsorption isotherm task within the Sorption module of Materials Studio software. The Universal Force Field (UFF) was chosen for the simulations because it produced adsorption isotherms that matched experimental data more closely than those generated using COMPASS, Dreiding, CVFF, and PCFF force fields, as shown in Figure S3. The van der Waals interactions were computed using the atom-based summation method with energy truncation via a cubic spline function (spline width: 1 Å) and a cut-off distance of 10 Å, which is sufficient for accurate results (validated in Figure S6) while remaining below half the unit cell length to prevent periodic self-interactions; electrostatic interactions were treated using the Ewald summation method. Atomic charges derived from DFT calculations were applied to both the molecules and zeolite frameworks, with the average values of charges used in the simulations listed in Table S3. The equilibrium and production steps were set to fine accuracy, with 10^5^ steps for equilibrium and 10^6^ steps for production, sufficiently large to balance computational accuracy and efficiency, as validated by testing (Figure S7). To ensure realistic adsorption behavior, the β-cages of the zeolites were blocked using inert He atoms. These served as non-interacting dummy atoms to prevent unphysical adsorption within inaccessible regions, thereby preserving the structural and functional integrity of the zeolite framework.

#### 2.3.3. Diffusion Behavior

The fixed-pressure adsorption task in the Sorption module of Materials Studio software was performed at 298 K and 200 kPa, representing the lower-temperature and higher-pressure conditions used in this study. The same force field and parameters used in the GCMC simulations (as in the previous section) were applied to determine the lowest energy configuration for CO_2_ adsorption. Under these conditions, 56 CO_2_ molecules were adsorbed in zeolite ITQ-29, and 77 CO_2_ molecules were adsorbed in zeolite 5A.

Subsequently, MD simulations were conducted in the Materials Studio software using the Forcite module on the lowest-energy configuration obtained from GCMC, with the zeolite framework treated as rigid and the CO_2_ molecules treated as fully flexible during the simulations. The system was equilibrated under the NVT ensemble (constant number of molecules, volume, and temperature) with a time step of 2.25 fs. Trajectory frames were recorded every 5000 steps, and the mean square displacement (MSD) was calculated at 10 ps intervals over a total simulation time of 1 ns.

The diffusion coefficient (*D*) of CO_2_ in zeolite 5A was determined by analyzing the MSD of CO_2_ molecules, as described by the following equations [56]:

(9) $$\mathrm{MSD}=\frac{1}{N_{m}N_{t}}\sum_{t}^{N_{t}}\sum_{i=1}^{N_{m}}{\left(\vec{r_{i}}\left(t\right)\;-\;\vec{r_{i}}\left(0\right)\right)}^{2}$$

(10) MSD = 6*Dt* + *C*

where *r_i_*(*t*) − *r_i_*(0) is the displacement of molecule *i* at time *t*, *N*_m_ is the number of adsorbate molecules, *N*_t_ is the number of independent trajectories, *C* is a constant for the ballistic regime of diffusion, and the diffusion coefficient is derived from the slope of the MSD versus time plot, where *D* equals one-sixth of the slope.

To evaluate temperature effects, MD simulations were performed at 298, 323, 348, and 373 K, with temperature control maintained using the Nose–Hoover thermostat to ensure system equilibrium.

## 3. Results and Discussion

### 3.1. Laboratory Experiments Output

#### 3.1.1. Characterization

**Figure 2:**
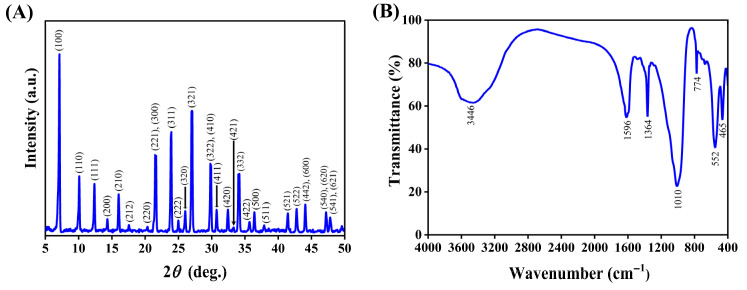
(**A**) XRD pattern of zeolite 5A, showing sharp peaks indicative of high crystallinity. (**B**) FTIR spectrum of zeolite 5A, displaying characteristic Si-O and Al-O vibrations that confirm the integrity of the zeolite 5A framework.

The XRD pattern in Figure 2A displays distinct diffraction peaks indexed to the (100), (110), (111), (210), (221), (331), (320), (321), (410), (411), (420), (332), (442), and (541) crystallographic planes at 2*θ* values of 7.14°, 10.09°, 12.36°, 15.97°, 21.49°, 23.77°, 25.86°, 26.85°, 29.65°, 30.53°, 32.23°, 33.84°, 44.20°, and 47.52. These peaks are consistent with the JCPDS reference PDF# 75-1151, confirming the phase purity and characteristic crystalline framework of zeolite 5A [57,58].

The FTIR spectrum of zeolite 5A (Figure 2B) reveals that a high specific surface area and high surface energy promote moisture adsorption, leading to the formation of hydroxyl groups (OH^−^) and multilayers of physically adsorbed water, as evidenced by the broad, intense peak at 3446 cm^−1^, corresponding to O–H stretching vibrations of adsorbed water [59]. The band at 1596 cm^−1^ is attributed to the O–H bending vibrations of adsorbed water molecules [60]. A band at 1364 cm^−1^ is assigned to carbonate (CO_3_)^2−^ ions, likely from atmospheric CO_2_ adsorption at the surface during sample handling [61,62]. The peak at 1010 cm^−1^ corresponds to the asymmetric stretching vibrations of the O–(Si, Al)–O linkages within the zeolite framework. The band at 774 cm^−1^ reflects Al-O tetrahedral stretching vibrations, while the peak at 552 cm^−1^ indicates the presence of secondary structural building units in the framework. The band at 465 cm^−1^ is assigned to the T-O bending vibrations (where T = Si or Al).

**Figure 3:**
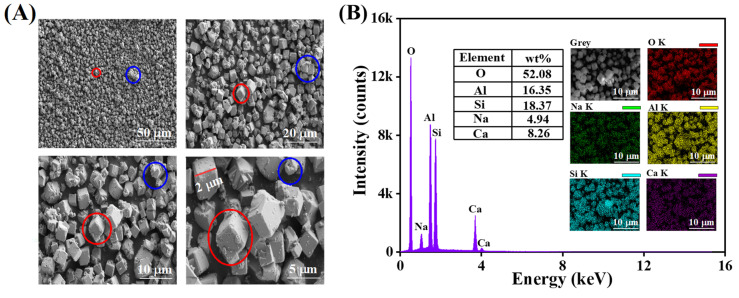
(**A**) Surface morphology of zeolite 5A. (**B**) EDS mapping and spectrum of zeolite 5A.

SEM observations and EDS elemental mapping were conducted to examine the surface morphology and elemental composition of zeolite 5A, as presented in Figure 3. In SEM images, zeolite 5A shows a cubic crystal structure with smooth surfaces and particle sizes below 2 µm (Figure 3A, red marks), along with circular features containing small pores (marked in blue), confirming a predominantly crystalline phase with minor amorphous content and a partially ordered zeolite framework. Figure 3B presents the EDS elemental mapping and spectrum of zeolite 5A, revealing a uniform elemental distribution, with oxygen (52.08 wt%), aluminum (16.35 wt%), silicon (18.37 wt%), sodium (4.94 wt%), and calcium (8.26 wt %).

**Figure 4:**
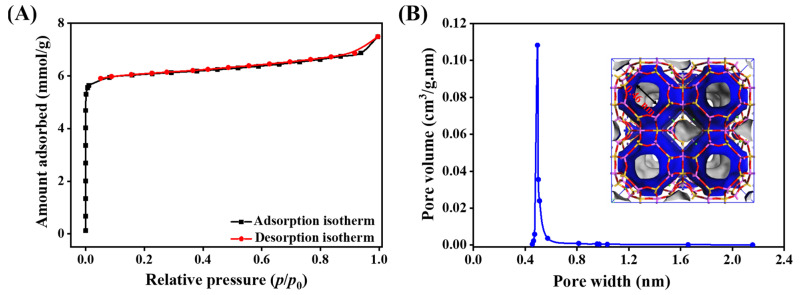
(**A**) N_2_ adsorption–desorption isotherm of zeolite 5A, showing typical microporous behavior. (**B**) Pore size distribution curve and structural model of zeolite 5A, illustrating the uniform microporous structure and crystalline framework.

The pore structure of zeolite 5A was characterized using N_2_ adsorption and desorption isotherms (Figure 4A), which exhibit a Type I isotherm of typical microporous materials. The Connolly surface simulation method was used to calculate the textural properties of zeolite 5A and to validate the model against experimental data. As presented in Figure 4B and Table 1, the simulated and experimental values show strong agreement for BET surface area (758 versus 743 m^2^/g), pore volume (0.260 versus 0.239 cm^3^/g), and average pore size (0.46 versus 0.47 nm), validating the reliability of the model and the structural characteristics of zeolite 5A. The effective pore size of zeolite exceeds the kinetic diameter of CO_2_ (0.33 nm), facilitating efficient diffusion and adsorption of CO_2_ molecules within microporous channels.

#### 3.1.2. Adsorption Isotherms

**Figure 5:**
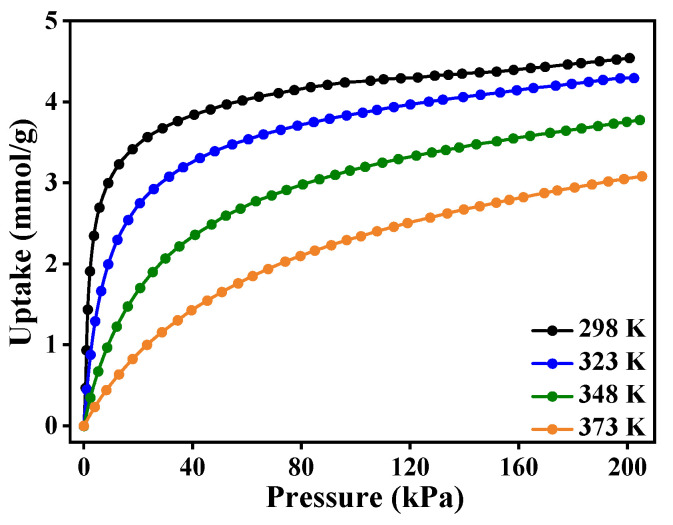
The adsorption isotherms of CO_2_ in zeolite 5A across varying temperatures.

The CO_2_ adsorption isotherms in zeolite 5A at different temperatures are shown in Figure 5. As temperature increases, the CO_2_ uptake decreases, indicating that lower temperatures are more favorable for CO_2_ adsorption. Specifically, the CO_2_ uptake at 298 K is the highest, reaching 4.53 mmol/g. The temperature influence on CO_2_ uptake demonstrates temperature-dependent behavior, where lower temperatures enhance adsorption due to reduced molecular motion and stronger adsorbent–adsorbate interactions, while higher temperatures increase thermal motion, weakening these interactions and leading to reduced uptake.

The CO_2_ uptake increases rapidly at low pressures due to the availability of abundant adsorption sites. As pressure increases, at approximately 20 kPa, CO_2_ adsorption increases sharply, indicating micropore filling and a strong interaction between CO_2_ and the zeolite framework. At higher pressures, the loadings approach equilibrium due to the progressive saturation of adsorption sites. Zeolite 5A contains exchangeable cations such as Ca^2+^ and Na^+^, which intensify the adsorbent–adsorbate interactions, enhancing the adsorption capacity.

The adsorption data were fitted using the Langmuir, Freundlich, Sips, and Toth models, as shown in Figure S8 (the data points represent the experimental results, while the black dashed lines correspond to the fitted isothermal models). Among these models, the Toth isotherm model provides the best fit, with a high coefficient of determination (adj. *R*^2^) of approximately 0.9999. The goodness of the fit with the Toth model suggests that the zeolite surface exhibits heterogeneous adsorption sites, indicating an adsorption mechanism with varying adsorption energies across the surface.

The parameter values derived from the Toth isotherm model, as shown in Table S4, indicate that both the maximum adsorption capacity (*q*_m_) and equilibrium constant (*k*_T_) decrease with increasing temperature, revealing that the adsorption process is more favorable at lower temperatures. The parameter *m* is less than 1, indicating a heterogeneous surface with varying adsorption energies, implying that the adsorption sites in zeolite 5A have different affinities for CO_2_, resulting in a mixed adsorption mechanism. Additionally, the lower Δ*Q* values derived from the Toth model further confirm the suitability of the model for describing CO_2_ adsorption in zeolite 5A. The separation factor (*R*_L_) calculated from the Toth isotherm equilibrium constant yields values below 1 at 298 K, as shown in Figure S9, confirming the thermodynamic favorability of the adsorption process and indicating strong adsorbate–adsorbent interactions.

#### 3.1.3. Adsorption Thermodynamics

**Figure 6:**
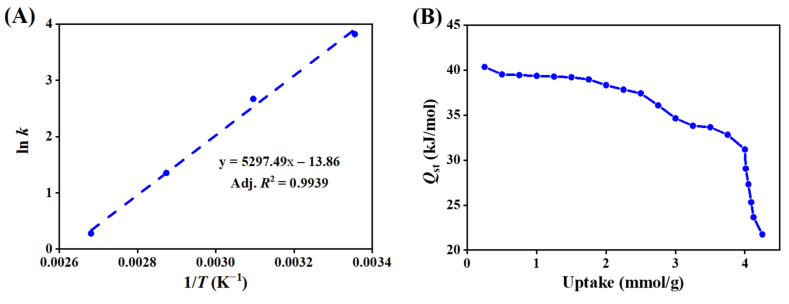
(**A**) The van’t Hoff plot showing the temperature dependence of the equilibrium constant for CO_2_ adsorption in zeolite 5A, where the negative slope confirms the exothermic nature of the process. (**B**) Isosteric heat of adsorption (*Q*_st_) as a function of CO_2_ uptake, showing a decreasing trend with increasing loading, attributed to the progressive occupation of lower-energy adsorption sites.

The thermodynamic parameters for CO_2_ adsorption in zeolite 5A were determined using the van’t Hoff equation based on the equilibrium constants obtained from the Toth isotherm, providing insight into the contributions of enthalpy and entropy to the adsorption process. A plot of ln(*k)* versus 1/*T*, shown in Figure 6A, yields a straight line with a slope of Δ*H*^0^/R and an intercept of Δ*S*^0^/R, from which the standard enthalpy (Δ*H*^0^) and entropy (Δ*S*^0^) changes were obtained. The standard Gibbs free energy change (Δ*G*^0^) was calculated to assess the spontaneity of the process at different temperatures. The resulting thermodynamic parameters are summarized in Table S5. The negative value of Δ*H*^0^ equal to −44.04 kJ/mol confirms the exothermic nature of CO_2_ adsorption, while the consistently negative Δ*G*^0^ values at 298–373 K indicate a spontaneous process [63,64]. The decrease in the magnitude of Δ*G*^0^ with increasing temperature suggests that adsorption becomes less favorable at higher temperatures. Moreover, the negative entropy change (Δ*S*^0^ = −115.23 J/mol·K) indicates reduced molecular randomness at the solid–gas interface, suggesting that CO_2_ molecules adopt a more ordered configuration upon adsorption, with minimal disruption to the zeolite 5A framework [65].

The isosteric heat of adsorption reflects the strength of interaction between CO_2_ molecules and the zeolite 5A surface. As shown in Figure 6B, the isosteric heat of adsorption gradually decreases with increasing CO_2_ uptake. This trend indicates that adsorption initially occurs at the most energetically favorable sites, which provide stronger interactions. As these high-energy sites become saturated, subsequent adsorption takes place on less active sites with weaker interactions, resulting in a lower isosteric heat of adsorption. This behavior reflects the relative uniformity of the zeolite 5A surface and the progressive occupation of sites with varying adsorption energies.

#### 3.1.4. Adsorption Kinetics

**Figure 7:**
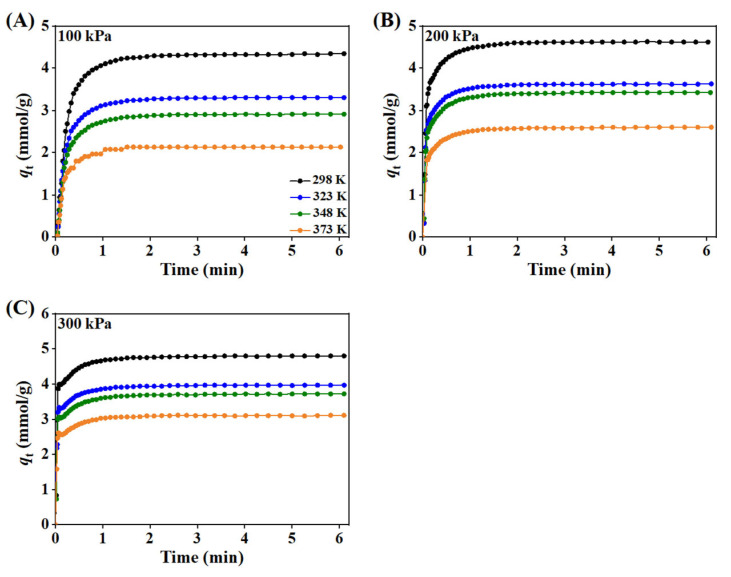
The adsorption kinetics of CO_2_ in zeolite 5A at different pressures and temperatures: (**A**) at 100 kPa, (**B**) 200 kPa, and (**C**) at 300 kPa.

The adsorption kinetics of CO_2_ in zeolite 5A at different temperatures and pressures are shown in Figure 7. At the initial stage of adsorption, the CO_2_ uptake rate is high due to the rapid occupation of abundant available sites on the zeolite surface, with strong interactions between CO_2_ and the zeolite framework accelerating the process. As adsorption proceeds, the adsorption rate slows down as the available active sites are gradually occupied, leading to lower affinity sites for CO_2_ to adsorb. Eventually, the system reaches equilibrium, where the amount of CO_2_ adsorbed becomes constant over time.

The temperature-dependent CO_2_ uptake over time, as shown in Figure 7A–C, demonstrates that higher temperatures reduce the overall adsorption capacity due to decreased affinity between CO_2_ molecules and the zeolite surface, whereas lower temperatures enhance CO_2_ interaction with the adsorbent, increasing uptake. Higher pressure increases CO_2_ concentration, enhancing molecular collisions with the zeolite surface, which in turn accelerates the uptake rate over time and results in a greater equilibrium adsorption capacity; this effect is more pronounced at lower temperatures, where adsorption is favored, and at higher pressures, where the driving force for mass transfer and site occupancy is increased.

To understand the adsorption process, three kinetics models were applied. The pseudo-first-order model (Equation (11)) [66] was used to describe physical adsorption, linking the adsorbed amount at any time to the equilibrium capacity. For systems dominated by chemisorption, the pseudo-second-order model (Equation (12)) [67] was employed, as it better fits cases where chemical interactions control the rate. Additionally, the intra-particle diffusion model (Equation (13)) [68] helped assess the diffusion of CO_2_ molecules within the zeolite pores. Experiments conducted at 100 kPa across different temperatures allowed for differentiation between surface reaction-controlled and diffusion-controlled mechanisms, clarifying the dominant adsorption pathway. The equations for these models are as follows:

(11) $$\ln\left(q_{e}-q_{t}\right)=\;\ln\;{(q}_{e})-\;k_{1}t$$

(12) $$\frac{t}{q_{t}}\;=\;\frac{1}{k_{2}q_{e}^{2}}\;+\;\frac{t}{q_{e}}$$

(13) $$q_{t}={\;K}_{\mathrm{diff}}{\;t}^{0.5}+C$$

where *q*_e_ and qt represent the amounts of CO_2_ that are adsorbed at equilibrium and at a specific time, respectively, measured in mmol/g. The rate constants for the different kinetics models are *k*_1_ (measured in min^–1^), *k*_2_ (measured in g/mmol·min), and *K*_diff_ (measured in mmol/g·min^1/2^). The constant *C* refers to the thickness of the boundary layer.

**Figure 8:**
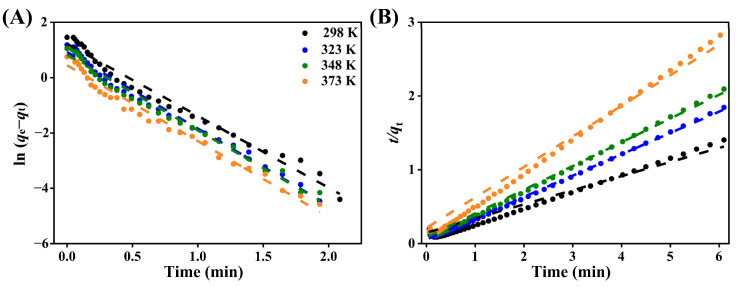
Linear dependence of the adsorption kinetics of CO_2_ in zeolite 5A at different temperatures (298–373 K): (**A**) the pseudo-first-order model, and (**B**) the pseudo-second-order model.

The pseudo-first-order model was analyzed by constructing a linear plot of ln(*q*_e_−*q*_t_) versus time, from which the slope and intercept were extracted. These values were then used to calculate the rate constant (*k*_1_) and the calculated equilibrium adsorption (*q*_e_) (Figure 8A). Similarly, a plot of *t*/*q*_t_ versus time (Figure 8B) was employed to derive the pseudo-second-order rate constant (*k*_2_) and the calculated equilibrium adsorption (*q*_e_) from the slope and intercept of the lines. The data in Figure 8 clearly demonstrate that CO_2_ adsorption is temperature-dependent, with an increase in temperature (ranging from 298–373 K) leading to faster adsorption rates, as reflected by steeper slopes in both kinetics models.

Table 2 summarizes the parameters of the related kinetics models with linear fitting. Among them, the pseudo-first-order model provides a better fit to the experimental sorption data than the pseudo-second-order model, as evidenced by its exceptionally high coefficient of determination (adj. *R*^2^). This strong correlation suggests that the pseudo-first-order model effectively captures the reaction mechanism governing CO_2_ adsorption in zeolite 5A. Across all adsorption conditions, the calculated CO_2_ uptake decreases with increasing temperature, consistent with the exothermic nature of physical adsorption [25]. According to the pseudo-first-order kinetics model, CO_2_ molecules adsorb onto the zeolite 5A surface through physical adsorption, governed by diffusion processes without chemical bonding. This process is driven by weak intermolecular forces, such as van der Waals interactions [69].

**Figure 9:**
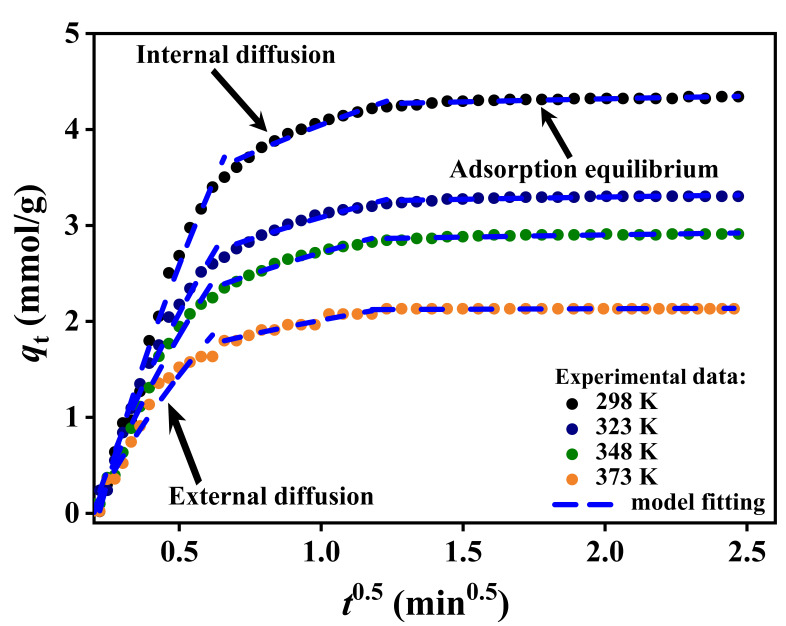
Intraparticle diffusion model for CO_2_ adsorption in zeolite 5A at various temperatures.

The intraparticle diffusion model was applied to assess CO_2_ adsorption kinetics mechanisms at varying temperatures, based on the assumption that the adsorption process is controlled by diffusion. The adsorption curves, as shown in Figure 9, exhibit a clear multi-linear trend with three distinct regions, corresponding to external film diffusion, intraparticle diffusion or internal mass transfer diffusion, and surface adsorption equilibrium. This confirms the presence of sequential diffusion steps controlling the process. The diffusion rate constant (*K*_diff_), boundary layer thickness (*C*), and coefficient of determination (adj. *R*^2^), derived from the slope and intercept of each region, are summarized in Table 3.

According to the diffusion rate, the adsorption process progresses through three phases characterized by distinct *K*_diff_ values. To identify the boundaries between these phases, the adsorption data were plotted as *q_t_* versus *t*^0.5^, and the curve was visually segmented based on noticeable changes in slope and curvature. Each segment showing linear behavior was then individually fitted using the intraparticle diffusion model. The start and end points of each region were determined by the extent of linearity and supported by high coefficients of determination (adj. *R*^2^), while the intersections of the fitted lines were used to define the transitions between diffusion regimes. In the first phase (*K*_diff1_), the high diffusion rate reflects rapid CO_2_ transport through the external film, indicating minimal resistance to mass transfer. In the second phase (*K*_diff2_), the diffusion rate decreases as molecular transport becomes controlled by movement within the internal pore structure of zeolite 5A. In the third phase (*K*_diff3_), the lowest diffusion rate is observed, corresponding to the approach of surface adsorption equilibrium, where the availability of active sites diminishes [70]. The overall decline in *K*_diff_ across the three phases (*K*_diff1_ > *K*_diff2_ > *K*_diff3_) demonstrates a gradual transition from fast external diffusion to increasingly restricted internal transport, ultimately leading to equilibrium conditions.

The activation energy (*E*ₐ) is an important factor in adsorption kinetics, representing the energy required for gas molecules to move through the adsorbent pores and reach the adsorption sites. A lower *E*ₐ indicates that the process requires less energy and occurs more easily, while a higher *E*ₐ suggests a more energy-demanding process, potentially indicating a chemical reaction. The activation energy for CO_2_ adsorption can be determined using the Arrhenius equation (Equation (14)) [71]:

(14) $$k_{\mathrm{eff}}=A\cdot \exp\left(-\frac{E_{a}}{RT}\right)$$

where *k*_eff_ represents the effective rate constant, *A* indicates a pre-exponential factor, *E*_a_ is the activation energy, R is the universal gas constant, and *T* is the temperature.

**Figure 10:**
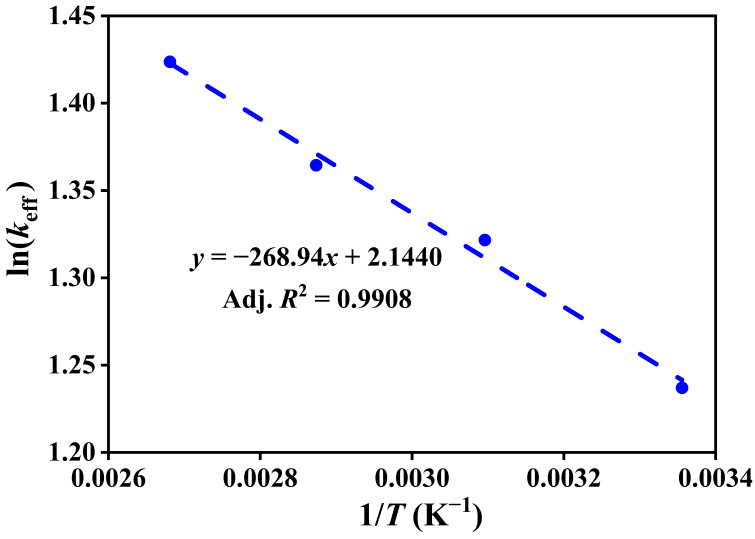
Arrhenius plot of ln(*k*_eff_) versus (1/*T*) for CO_2_ adsorption in zeolite 5A. The activation energy is determined from the slope of the linear dependence.

The effective rate constants (*k*_eff_) at each temperature were determined by fitting the experimental adsorption kinetics data (*q_t_* versus *t*) to the pseudo-first-order kinetics model using nonlinear regression. These values were used to construct the Arrhenius plot, where ln(*k*_eff_) was plotted against the reciprocal temperature (1/*T*), as shown in Figure 10. The resulted strong linear relationship confirms that the adsorption process follows Arrhenius behavior. The activation energy (*E*_a_) was calculated from the slope of the fitted line, indicating the temperature sensitivity of the adsorption rate and reflecting the combined influence of mass transfer and surface reaction mechanisms. The *E*_a_ calculated from the slope is 2.24 kJ/mol, indicating a low energy barrier for the adsorption process, which suggests that physical diffusion governs the process rather than chemical reaction, making it relatively insensitive to temperature changes. The high coefficient of determination (adj. *R*^2^) further confirms the reliability of the fitting and indicates process efficiency and stability across a range of temperatures.

### 3.2. Simulation Techniques Output

In addition to the lab experiments of CO_2_ adsorption in zeolite 5A, the DFT calculations and molecular simulations were conducted for zeolite ITQ-29 and zeolite 5A to quantify CO_2_–adsorbent interactions and reveal molecular-level dynamics and spatial distribution within the porous structure.

#### 3.2.1. Adsorption Structures and Adsorption Energies

**Figure 11:**
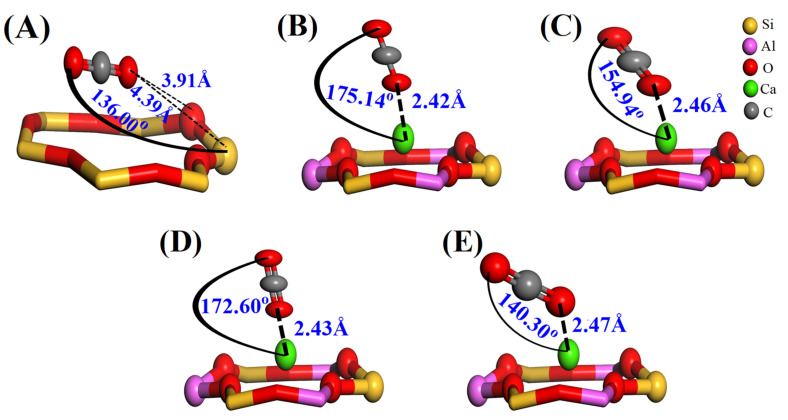
CO_2_ adsorption configurations after geometry optimization on six-membered rings of LTA zeolites. (**A**) Zeolite ITQ-29, where the CO_2_ molecule moves away from the O and Si atoms in the six-membered ring, indicating weak adsorption; (**B**,**C**) zeolite 5A, resulting from initially horizontal CO_2_ configurations, with angular changes demonstrating the interaction with Ca^2+^ cation; (**D**,**E**) zeolite 5A, resulting from initially vertical CO_2_ configurations, with angular changes indicating interaction with Ca^2+^ cations. Spheres: yellow (Si), red (O), pink (Al), green (Ca), and grey (C).

The adsorption structures of CO_2_ in zeolite ITQ-29 and zeolite 5A are shown in Figure 11, which were obtained through geometry optimization using DFT calculations based on the initial configurations depicted in Figure S5.

A comparative analysis of CO_2_ adsorption in zeolite ITQ-29 and zeolite 5A reveals distinct interaction mechanisms driven by the presence or absence of extraframework cations. In zeolite ITQ-29, the geometry optimization results shown in Figure 11A revealed that the initial uniform distance of 1.27 Å, determined before optimization in Figure S5A, between the O atom of the CO_2_ molecule and the nearest framework O and Si atoms in the six-membered ring increased to 3.91 Å (O···O) and 4.39 Å (O···Si), respectively, indicating the formation of a physisorbed state characterized by weak van der Waals and electrostatic interactions with the silicate framework.

In zeolite 5A, the initial distance between the CO_2_ molecule and the Ca^2+^ cation was fixed at 2.66 Å for all configurations, as shown in Figure S5B–E. The CO_2_ molecule was initially positioned in either a horizontal orientation (C atom facing Ca^2+^) or a vertical orientation (O atom facing Ca^2+^). After geometry optimization (Figure 11B–E), the Ca···CO_2_ distances decreased to 2.42, 2.46, 2.43, and 2.47 Å, respectively. The corresponding bond angles between Ca^2+^ and CO_2_ are 175.14°, 154.94°, 172.60°, and 140.30°, reflecting deviations from the ideal linear (180°) or perpendicular (90°) orientations. Notably, configurations initially in a horizontal orientation are reoriented during optimization, with the Ca^2+^ cation preferentially binding to one of the oxygen atoms of CO_2_ rather than the carbon. This reorientation indicates the electrostatic preference of Ca^2+^ for the more electronegative oxygen atoms, reinforcing the strong, directionally dependent interactions between CO_2_ and Ca^2+^ sites in zeolite 5A.

The adsorption energies of CO_2_ in zeolite ITQ-29 and zeolite 5A are summarized in Table 4. In zeolite 5A, CO_2_ exhibits significantly more negative adsorption energies compared to zeolite ITQ-29, indicating stronger interactions. For zeolite ITQ-29, as shown in configuration Figure 11A, the adsorption energy is only −12.16 kJ/mol. In contrast, the adsorption energies for configurations depicted in Figure 11B–E are −46.65, −45.78, −47.26, and −48.85 kJ/mol, respectively. The computed adsorption energies show good agreement with the experimentally measured adsorption enthalpies for zeolite 5A obtained in this study and are consistent with the literature values reported for both zeolites ITQ-29 and 5A [68,72,73], further supporting the reliability of the computational approach. The adsorption energies of CO_2_ on various cation-exchanged sites indicate a clear distinction between monovalent and divalent cations. As shown in Table S6, monovalent cations such as Na^+^ and K^+^ exhibit lower adsorption energies, while divalent cations like Ca^2+^ and Mg^2+^ demonstrate significantly stronger interactions with CO_2_. This suggests that CO_2_ preferentially adsorbs at high-affinity sites associated with divalent cations, such as Ca^2+^, over lower-affinity sites like Na^+^.

In the deformation charge density map (Figure S10A), the red regions indicate electron accumulation, and the blue regions represent electron depletion. The charge redistribution features are absent in ITQ-29 (Figure S10(A1)), which shows more localized electron density around the CO_2_ molecule. In zeolite 5A (Figure S10(A2)), strong electron accumulation is observed near the Ca^2+^ and CO_2_ oxygen atoms, along with electron depletion around the framework, indicating pronounced polarization and partial charge transfer between the framework and the adsorbed CO_2_. The Mulliken charge analysis supports these observations, showing that the oxygen atom in zeolite 5A reaches −0.372 compared to −0.277 in ITQ-29, reflecting enhanced polarization in zeolite 5A. The partial density of states (PDOS) further supports this, showing broader overlap between CO_2_ 2p and Ca^2+^ 4s states in 5A (Figure S10(B2)), whereas no overlap is observed in ITQ-29 (Figure S10(B1)). These results confirm that zeolite 5A enables stronger electronic coupling and more favorable CO_2_ adsorption than ITQ-29.

#### 3.2.2. Adsorption Isotherm Simulations

**Figure 12:**
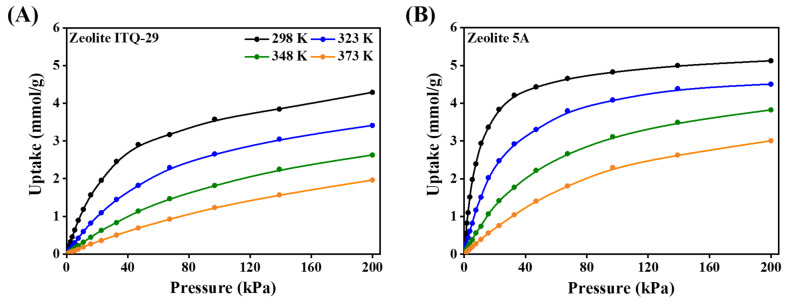
CO_2_ adsorption isotherms at different temperatures in (**A**) zeolite ITQ-29 and (**B**) zeolite 5A, obtained using Grand Canonical Monte Carlo simulations.

The isotherms of CO_2_ uptake in zeolite ITQ-29 and zeolite 5A at various temperatures, obtained from molecular simulations using Grand Canonical Monte Carlo (GCMC), are shown in Figure 12. CO_2_ uptake decreases with increasing temperature in both zeolites, indicating that lower temperatures favor adsorption. In zeolite ITQ-29 (Figure 12A), the CO_2_ uptake is lower than in zeolite 5A, and the adsorption progresses gradually with pressure, without reaching clear equilibrium at the final pressure, suggesting weaker interactions and slower site saturation. In contrast, zeolite 5A (Figure 12B) shows a sharp increase in CO_2_ uptake at low pressures due to abundant adsorption sites, followed by a plateau at higher pressures as these sites become saturated. The higher uptake in zeolite 5A is attributed to the presence of Ca^2+^ and Na^+^ cations, which enhance electrostatic interactions with CO_2_ molecules. Zeolite ITQ-29, which lacks extraframework cations, adsorbs CO_2_ mainly through weak van der Waals forces. These observations indicate the essential role of cations in promoting stronger adsorbate–adsorbent interactions and achieving higher CO_2_ uptake.

In this work, the simulation results for zeolite 5A were validated by the laboratory measurements (Figure S11). There is good agreement between simulation and experimental results across the studied temperature and pressure ranges, confirming the reliability and accuracy of the selected simulation parameters. Moreover, the use of the UFF in combination with GCMC simulations effectively captures the CO_2_ adsorption behavior in both zeolites ITQ-29 and 5A.

**Figure 13:**
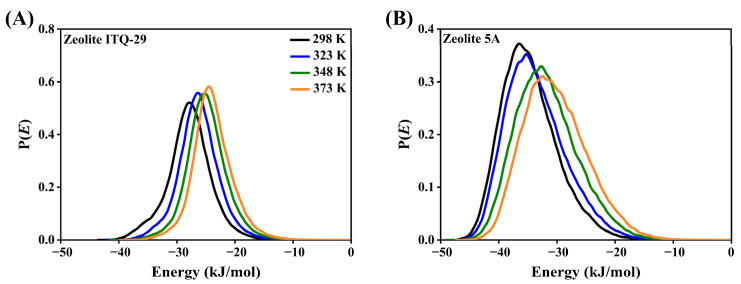
Energy distribution profiles for CO_2_ adsorption in (**A**) zeolite ITQ-29 and (**B**) zeolite 5A at different temperatures. The *y*-axis represents the probability density, P(*E*) (a.u.), normalized to unity. More negative energy values indicate stronger CO_2_–adsorbent interactions.

The energy distribution profiles for CO_2_ adsorption in zeolites ITQ-29 and 5A at various temperatures are shown in Figure 13. The profile for ITQ-29, as shown in Figure 13A, is relatively narrow and symmetric, indicating predominantly uniform adsorption sites consistent with the same strength. In contrast, the broader and asymmetric profile observed for zeolite 5A, as shown in Figure 13B, reveals the presence of multiple adsorption sites with different strengths, reflecting heterogeneous adsorption behavior.

As temperature increases, the energy distribution curves shift toward less negative values, consistent with the decrease in adsorption strength and the temperature-dependent adsorption isotherms, which demonstrate that lower temperatures are more favorable for CO_2_ adsorption, as illustrated in Figure 13. To compare zeolites ITQ-29 and 5A, interaction energies for zeolite ITQ-29 are less negative, indicating weaker CO_2_ interactions. In contrast, zeolite 5A exhibits more negative interaction energies, reflecting stronger interactions due to the presence of Ca^2+^ and Na^+^ cations. These cations enhance the strength of electrostatic interactions with CO_2_ molecules, whereas zeolite ITQ-29 depends on weaker van der Waals interactions.

Figure S12 presents the isosurface energy fields of CO_2_ within the frameworks of zeolite ITQ-29 and zeolite 5A, illustrating the spatial distribution of the CO_2_–framework interaction energies. An isosurface energy field is a three-dimensional map where each surface connects points in space with the same interaction energy, enabling the identification of regions corresponding to different adsorption sites. The color scheme represents the spatial distribution of CO_2_ molecules using three distinct colors: red, blue, and green. In Figure S12A, corresponding to zeolite ITQ-29, the isosurface regions appear fully connected and uniformly distributed throughout the framework. This reflects predominantly uniform adsorption sites with similar strengths, consistent with the symmetric adsorption profile seen in Figure 13A. As ITQ-29 lacks extraframework cations, the interaction with CO_2_ is governed mainly by weak van der Waals forces, which also explains the homogeneous distribution of colors in the regions. In contrast, Figure S12B, representing zeolite 5A, shows blue and green isosurface regions that are partially connected, with some areas linked while others remain disconnected or loosely connected. This indicates the presence of multiple adsorption sites with different strengths, consistent with the broader and asymmetric adsorption profile observed in Figure 13B. Compared to the fully connected and uniformly distributed regions in Figure S12A, this partial connectivity reflects a varied distribution of adsorption sites with different strengths. Within the overall context of Figure S12, these differences indicate the uneven spatial arrangement of adsorption sites in zeolite 5A, likely influenced by the presence of extraframework cations.

#### 3.2.3. Diffusion Coefficients and Activation Energies

**Figure 14:**
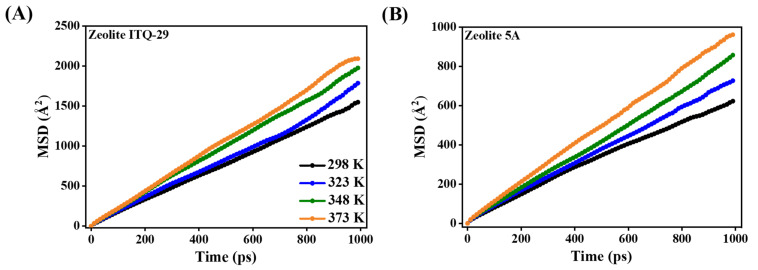
The mean square displacement (MSD) of CO_2_ molecules in (**A**) zeolite ITQ-29 and (**B**) zeolite 5A as a function of time at varying temperatures.

The diffusion behavior of CO_2_ in zeolite ITQ-29 and zeolite 5A as a function of MSD is shown in Figure 14, demonstrating that CO_2_ diffusion in both zeolites is highly sensitive to temperature, with increasing thermal energy significantly increasing molecular diffusion. This is reflected in the increasing MSD of CO_2_ molecules over time, indicating faster diffusion at higher temperatures [74].

Zeolite ITQ-29 (Figure 14A) exhibits higher MSD values compared to zeolite 5A (Figure 14B), primarily due to differences in framework interactions. The absence of framework cations minimizes electrostatic and van der Waals forces, allowing CO_2_ molecules to diffuse with less restriction. In contrast, zeolite 5A contains cations such as Ca^2+^, which create stronger adsorption sites and reduce diffusion, thereby increasing energy barriers for molecular displacement.

**Figure 15:**
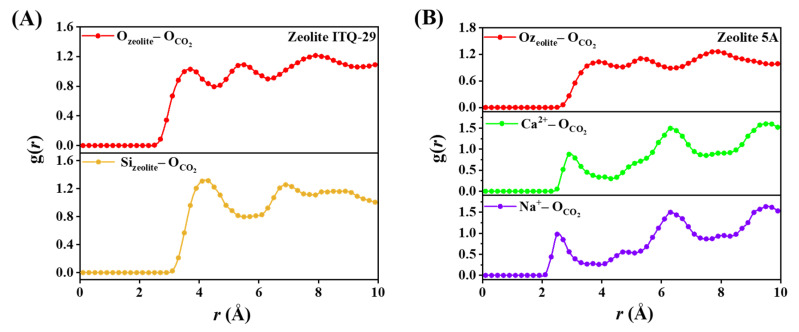
Radial distribution function (RDF) profiles of the O atom in CO_2_ with framework atoms and extraframework cations: (**A**) Zeolite ITQ-29, showing O_zeolite_–O_CO2_ and Si_zeolite_–O_CO2_ spatial distances; (**B**) zeolite 5A, showing O_zeolite_–O_CO2_, Ca^2+^–O_CO2_, and Na^+^–O_CO2_ spatial distances at 298 K.

The radial distribution function (RDF) analysis of the O atom in CO_2_ at the optimal temperature for adsorption (298 K) shows the spatial distribution relative to Si and O atoms in the framework of zeolite ITQ-29 and relative to O, Ca^2+^, and Na^+^ in zeolite 5A, as presented in Figure 15.

The RDF analysis in Figure 15A for CO_2_ distribution in zeolite ITQ-29 shows spatial correlations between the oxygen atoms of CO_2_ and the framework atoms, with peaks at approximately 3.7, 5.5, and 7.9 Å for O_zeolite_–O_CO2_ and at 4.3, 6.9, and 8.1 Å for Si_zeolite_–O_CO2_. These distances correspond to relatively weak interactions governed by van der Waals forces. In contrast, the RDF analysis in Figure 15B for zeolite 5A shows more distinct and localized correlations between CO_2_ and extraframework cations. For O_zeolite_–O_CO2_, peaks appear at approximately 3.9, 5.3, and 7.9 Å. The RDFs for Ca^2+^–O_CO2_ and Na^+^–O_CO2_ show prominent peaks at 2.9, 5.1, 6.3, and 9.5 Å and 2.7, 5.3, 6.3, and 9.5 Å, respectively. These observations indicate stronger electrostatic interactions modeled between the negatively charged oxygen atoms of CO_2_ and the positively charged extraframework cations, which contribute to CO_2_ adsorption and positioning within the zeolite pores.

**Figure 16:**
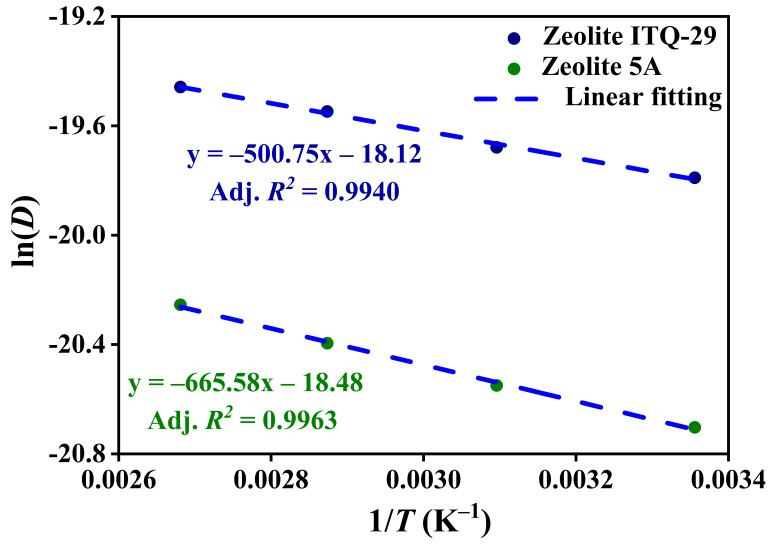
Arrhenius plots of ln(*D) *versus 1/*T*, where the activation energies are determined from the slopes of the linear regression.

The diffusion coefficients (*D*) as a function of temperature, along with Arrhenius plots of ln(*D*) versus inverse temperature (1/*T*) for CO_2_ in zeolites ITQ-29 and 5A, are presented in Figure S13 and Figure 16, respectively. The diffusivities of CO_2_ in zeolites ITQ-29 and 5A were calculated as one-sixth using the slope of the MSD versus time, resulting in the temperature-dependent values shown in Figure S13. The CO_2_ diffusivities at lower temperatures are lower than at higher temperatures for both zeolites. This reflects the thermally activated nature of diffusion; at lower temperatures, CO_2_ molecules have less kinetic energy and reveal stronger adsorption, leading to lower diffusivity. As temperature increases, molecular diffusion increases, resulting in higher diffusion coefficients. At 298 K, the diffusion coefficients are 2.54 × 10^–9^ m^2^/s for ITQ-29 and 1.02 × 10^–9^ m^2^/s for 5A, increasing to 3.54 × 10^–9^ m^2^/s and 1.59 × 10^–9^ m^2^/s at 373 K, respectively. The more pronounced increase observed for ITQ-29 suggests its pore structure facilitates the molecular diffusion of CO_2_ more than zeolite 5A.

In Figure 16, the activation energy for CO_2_ diffusion is lower in zeolite ITQ-29 (4.16 kJ/mol) than in zeolite 5A (5.54 kJ/mol), indicating reduced transport barriers in the purely siliceous framework. In zeolite 5A, the presence of Ca^2+^ and Na^+^ cations introduces strong electrostatic fields that impede molecular diffusion, whereas the structure of ITQ-29 allows more facile diffusion governed by weak van der Waals interactions. The 1.38 kJ/mol (33%) difference quantifies the additional energetic constraint imposed by cationic sites. These results underscore the role of framework composition and extraframework species in modulating diffusion behavior, offering a strategy for tuning zeolite performance in separation and storage applications.

Despite the ~35 kJ/mol difference in CO_2_ adsorption energies between ITQ-29 and zeolite 5A, the diffusion activation energy differs by only 1.38 kJ/mol. This discrepancy arises because adsorption energy reflects the strength of molecule-framework interactions, while diffusion activation energy depends on the energy barriers between adjacent adsorption sites. In zeolite 5A, stronger adsorption does not necessarily lead to proportionally higher diffusion barriers. Although ITQ-29 has smaller cavities, its pore structure may offer smoother diffusion paths, resulting in comparable or lower diffusion barriers.

## 4. Conclusions

The adsorption of carbon dioxide in LTA-type zeolites was investigated based on integrating experimental and simulation techniques. Structural characterization verified the crystalline integrity and pore accessibility of the zeolites. The experimental measurement results confirmed that the adsorption of zeolite 5A for CO_2_ is spontaneous, exothermic, and governed by physical interactions. The isosteric heat of CO_2_ adsorption decreases with increasing coverage, showing heterogeneous interactions at different sites like Ca^2+^ and Na^+^. The adsorption kinetics follow a pseudo-first-order model, which confirms a physisorption mechanism. The intraparticle diffusion model indicates that internal diffusion is the rate-limiting step. According to the DFT calculations, CO_2_ adsorption energy in zeolite 5A is more negative than in zeolite ITQ-29. The enhanced adsorption capability arises from stronger interactions between CO_2_ molecules and exchangeable cations, particularly Ca^2+^, facilitating the hybridization of their atomic orbitals. Molecular dynamics simulations further showed that CO_2_ molecules diffuse more rapidly in ITQ-29, where weaker van der Waals forces prevail, while zeolite 5A provides stronger adsorption but reduced diffusivity due to the enhanced electrostatic interactions with Ca^2+^/Na^+^. The 33% difference in activation energy between two LTA-type zeolites confirms the role of cationic sites in diffusion kinetics. Overall, zeolite 5A exhibits a favorable balance between CO_2_ uptake and retention, making it a promising candidate for gas separation applications. The integration of experimental observations and molecular simulations offers key insights into how framework composition and cation selection influence adsorption behavior, guiding the development of efficient zeolite-based CO_2_ capture systems.

## Figures and Tables

**Table 1:** Measured and simulated structural parameters (surface area, pore volume, and pore size) of zeolite 5A.

Zeolite 5A	BET Surface Area (m^2^/g)	Pore Volume (cm^3^/g)	Average Pore Width (nm)
Measured	743	0.239	0.47
Simulated	758	0.260	0.46

**Table 2:** Equilibrium loadings, rate constants, and correlation coefficients of CO_2_ adsorption in zeolite 5A at different temperatures for the pseudo-first-order and pseudo-second-order models.

Temperature (K)	Pseudo-First-Order			Pseudo-Second-Order		
	*q*_e_ (mmol/g)	*k*_1_ (min^−1^)	Adj. *R*^2^	*q*_e_ (mmol/g)	*k*_2_ (g/mmol·min)	Adj. *R*^2^
298	3.39	2.08	0.978	5.22	0.25	0.509
323	2.53	2.43	0.986	3.46	0.82	0.995
348	2.28	2.77	0.983	3.10	1.23	0.983
373	1.56	3.04	0.978	2.41	1.53	0.827

**Table 3:** The diffusion kinetics parameters for CO_2_ adsorption in zeolite 5A.

Temperature (K)	External Film Diffusion			Intraparticle Diffusion			Surface Adsorption at Equilibrium		
	*K*_diff1_	*C*	Adj. *R*^2^	*K*_diff2_	*C*	Adj. *R*^2^	*K*_diff3_	*C*	Adj. *R*^2^
298	10.75	−5.02	0.988	2.26	1.79	0.960	0.167	4.084	0.806
323	7.79	−3.43	0.980	1.70	1.38	0.956	0.126	3.119	0.738
348	7.08	−3.16	0.982	1.73	0.99	0.967	0.116	2.728	0.743
373	5.35	−2.34	0.961	1.14	0.87	0.935	0.027	2.094	0.937

**Table 4:** Adsorption energies/enthalpies of CO_2_ in zeolite ITQ-29 and zeolite 5A.

Adsorbent	Adsorption Energy/Enthalpy (kJ/mol)	Source
Zeolite ITQ-29	−12.16	This work
Zeolite 5A	−47.13 (average)	This work
Zeolite 5A	−44.04	This work (experiment)
Zeolite ITQ-29	−20.00	[72]
Zeolite 5A	−45.00	[73]
Zeolite 5A	−45.20	[68]

## Data Availability

The raw data supporting the conclusions of this article will be made available by the authors upon request.

## Supplementary Materials

The following supporting information can be downloaded at https://www.mdpi.com/article/10.3390/nano15141077/s1: Figure S1: Oblique view of the crystallographic structure of zeolite 5A. Spheres: yellow (Si), red (O), pink (Al), purple (Na^+^), and green (Ca^2+^); Figure S2: X-ray diffraction patterns of (A) zeolite ITQ-29 and (B) zeolite 5A; Figure S3: Isotherms of CO_2_ adsorption in zeolite 5A with different force fields and a cut-off distance of 12 Å at (A) 298 K and (B) 323 K; Figure S4: Isotherms of CO_2_ adsorption in zeolite 5A with different charges at (A) 298 K and (B) 323 K, where multiple charge sets were studied for CO_2_, while the zeolite 5A structure employed charges derived from Hirshfeld population analysis; Figure S5: Initial configurations of CO_2_ before geometry optimization on the surface of zeolite LTA. (A) Zeolite ITQ-29 with the CO_2_ molecule positioned near O and Si atoms in a six-membered ring. (B, C) Horizontal configurations with the carbon atom of CO_2_ placed near the Ca^2+^ cation, and (D, E) vertical configurations with the oxygen atom of CO_2_ placed near the Ca^2+^ cation; Figure S6: Isotherms of CO_2_ adsorption in zeolite 5A with different cut-off distances at (A) 298 K and (B) 323 K, where Muliken charge was applied for CO_2_, and the zeolite 5A structure employed charges derived from Hirshfeld population analysis; Figure S7: Convergence testing of equilibration and production steps at (A) 298 K and (B) 323 K, demonstrating the balance between computational accuracy and efficiency, with 10^5^ steps for equilibrium and 10^6^ steps for production (fine accuracy); Figure S8: Adsorption isotherms of CO_2_ in zeolite 5A at varying temperatures, fitted with (A) Langmuir, (B) Freundlich, (C) Sips, and (D) Toth models; Figure S9: Separation factor *R*_L_ for CO_2_ adsorption in zeolite 5A is < 1, indicating favorable adsorption; Figure S10. (A1, A2) deformation charge density maps and (B1, B2) partial density of states (PDOS) for CO_2_ adsorption in zeolites ITQ-29 and 5A. A1 and B1 correspond to zeolite ITQ-29, while A2 and B2 correspond to zeolite 5A. The region colors of red and blue indicate electron enrichment and depletion region, respectively, and the numbers near the atoms are Mulliken charges. The Fermi level is set to 0 eV in the PDOS analysis; Figure S11: Comparison of simulation and experimental CO_2_ adsorption data in zeolite 5A; Figure S12: Isosurface energy fields of CO_2_ adsorption in zeolites ITQ-29 and 5A. (A) Zeolite ITQ-29; broad range of interaction strengths (blue: strong, green: moderate, and red: weak). (B) Zeolite 5A; only strong to moderate interactions (blue and green) due to framework cations; Figure S13. Diffusion coefficients as a function of temperature; Table S1: Atomic coordinates of zeolite ITQ-29; Table S2: Atomic coordinates of zeolite 5A; Table S3: Average atomic charges derived from DFT calculations for CO_2_ molecules and LTA zeolites used in the simulations; Table S4: Adsorption parameters of the Toth model for CO_2_ adsorption in zeolite 5A; Table S5: Thermodynamic parameters of CO_2_ adsorption in zeolite 5A; Table S6: Adsorption energies of CO_2_ on different cation-exchanged sites.

## Abbreviations

The following abbreviations are used in this manuscript:

AC	activated carbon
MOFs	metal organic frameworks
COF	covalent organic frameworks
XRD	X-ray diffraction
FTIR	Fourier transform infrared spectroscopy
SEM	scanning electron microscope
EDS	energy-dispersive X-ray spectroscopy
DFT	density functional theory
GCMC	Grand Conical Monto Carlo
MD	molecular dynamics
*D*	diffusion coefficient
*E*_a_	activation energy
