# Supplementary material for: Insights into the Adsorption of Carbon Dioxide in Zeolites ITQ-29 and 5A Based on Kinetic Measurements and Molecular Simulations
Source: Nanomaterials (Basel). 2025 Jul 11;15(14):1077. doi: 10.3390/nano15141077 (PMC12300413; doi:10.3390/nano15141077)
Supplement: Supplementary file 1 [file nanomaterials-15-01077-s001.zip › nanomaterials-3725842-supplementary.pdf]

Supporting information

Insights into the Adsorption of Carbon Dioxide in Zeolites ITQ-29 and 5A Based on Kinetic Measurements and Molecular Simulations

Magdy Abdelghany Elsayed [1,2], Shixue Zhou [3,*], Xiaohui Zhao [1], Gumawa Windu Manggada [1], Zhongyuan Chen [3], Fang Wang [1], Zhijuan Tang [1],

[1]*College of Energy and Mining Engineering, Shandong University of Science and Technology, Qingdao 266590, China; magdyabdelghany10@163.com (M.A.E.); 18535824890@163.com (X.Z.); gumawawindu@gmail.com (G.W.M.); 13156213199@163.com (F.W.); 18791101514@163.com (Z.T.)*

[2]*Department of Mining and Petroleum Engineering, Faculty of Engineering, Al-Azhar University, Cairo 11884, Egypt*

[3]*College of Chemical and Biological Engineering, Shandong University of Science and Technology, Qingdao 266590, China; chenzhongyuan299@163.com*

**Correspondence: zhoushixue66@163.com*

Supporting Figures

Figure S1. Oblique view of the crystallographic structure of zeolite 5A. Spheres: yellow (Si), red (O), pink (Al), purple (Na^+), and green (Ca^{2+}).

Figure S2. X-ray diffraction patterns of zeolite ITQ-29 (A) and zeolite 5A (B).

Figure. S3. Isotherms of CO_2 adsorption in zeolite 5A with different force fields and a cut-off distance of 12 Å at (A) 298 K and (B) 323 K.

Figure. S4. Isotherms of CO_2 adsorption in zeolite 5A with different charges at (A) 298 K and (B) 323 K, where multiple charges sets were studied for CO_2, while the zeolite 5A structure employed charges derived from Hirshfeld population analysis.

Figure. S5. Initial configurations of CO_2 before geometry optimization on the surface of zeolite LTA. (A) Zeolite ITQ-29 with the CO_2 molecule positioned near O and Si atoms in a six-membered ring. (B, C) Horizontal configurations with the carbon atom of CO_2 placed near Ca^{2+} cation.

Figure. S6. Isotherms of CO_2 adsorption in zeolite 5A with different cut of distance at (A) 298 K and (B) 323 K, where Muliken charge was applied for CO_2, and the zeolite 5A structure employed charges derived from Hirshfeld population analysis.

Figure. S7. Convergence testing of equilibration and production steps at (A) 298 K and (B) 323 K, demonstrating the balance between computational accuracy and efficiency, with 10^5 steps for equilibrium and 10^6 steps for production (fine accuracy).

Figure. S8. Adsorption isotherms of CO_2 in zeolite 5A at varying temperatures, fitted with (A) Langmuir, (B) Freundlich, (C) Sips, and (D) Toth models.

Figure. S9. Separation factor R_L for CO_2 adsorption in zeolite 5A is < 1, indicating favorable adsorption.

Figure S10. (A1, A2) Deformation charge density maps and (B1, B2) partial density of states (PDOS) for CO_2 adsorption in zeolites ITQ-29 and 5A. A1 and B1 correspond to zeolite ITQ-29, while A2 and B2 correspond to zeolite 5A. The region colors of red and blue indicate electron enrichment and depletion region, respectively, and the numbers near the atoms are Mulliken charges. The Fermi level is set to 0 eV in the PDOS analysis.

Figure. S11. Comparison of simulation and experimental CO_2 adsorption data in zeolite 5A.

Figure. S12. Isosurface energy fields of CO_2 adsorption in zeolites ITQ-29 and 5A. (A) Zeolite ITQ-29; broad range of interaction strengths (blue: strong, green: moderate, red: weak). (B) Zeolite 5A; only strong to moderate interactions (blue and green) due to framework cations.

Figure S13. Diffusion coefficients as a function of temperature.

Supporting Tables

Table S1 Atomic coordinates of zeolite ITQ-29.

Table S2 Atomic coordinates of zeolite 5A.

Table S3 Average atomic charges derived from DFT calculations for CO_2 molecules and LTA zeolites used in the simulations.

Table S4 Adsorption parameters of the Toth model for CO_2 adsorption in zeolite 5A.

Table S5 Thermodynamic parameters of CO_2 adsorption in zeolite 5A.

Table S6 Adsorption energies of CO_2 on different cation-exchanged sites.

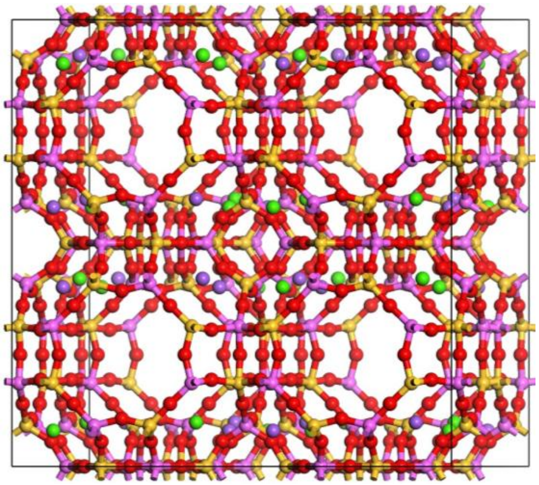

Figure S1. Oblique view of the crystallographic structure of zeolite 5A. Spheres: yellow (Si), red (O), pink (Al), purple (Na^+), and green (Ca^{2+}).

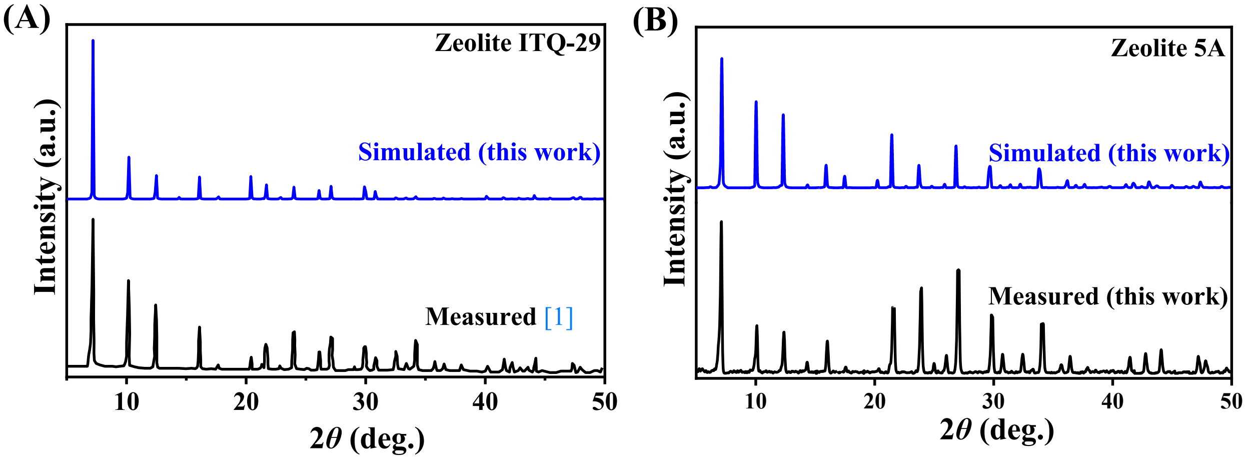

Figure S2. X-ray diffraction patterns of (A) zeolite ITQ-29 and (B) zeolite 5A.

To obtain the XRD patterns, Cu Kα radiation (λ = 1.541838 Å) was employed, with a scan range from 5–50° (2θ) and a step size of 0.05°. The diffraction peak profiles were fitted using a Pseudo-Voigt function, with the coefficients NA and NB fixed at 0.5 and 0.0, respectively. Instrumental broadening was taken using the Caglioti parameters, where U, V, and W were set to 0.02, −0.01, and 0.02$^{\circ 2}$, respectively, ensuring accurate modeling of the peak shapes. The peaks of the simulated X-ray diffraction patterns agree with the experimental measurements, with zeolite ITQ-29 data from the literature [1] and zeolite 5A obtained experimentally, demonstrating the accuracy and reliability of the cell models.

[1] Tarach, K.A.; Valencia, S; Góra-Marek, K.; Rey, F. Hierarchization of pure silica LTA zeolite. 16th International Conference on Environmental Science and Technology 2019, 920.

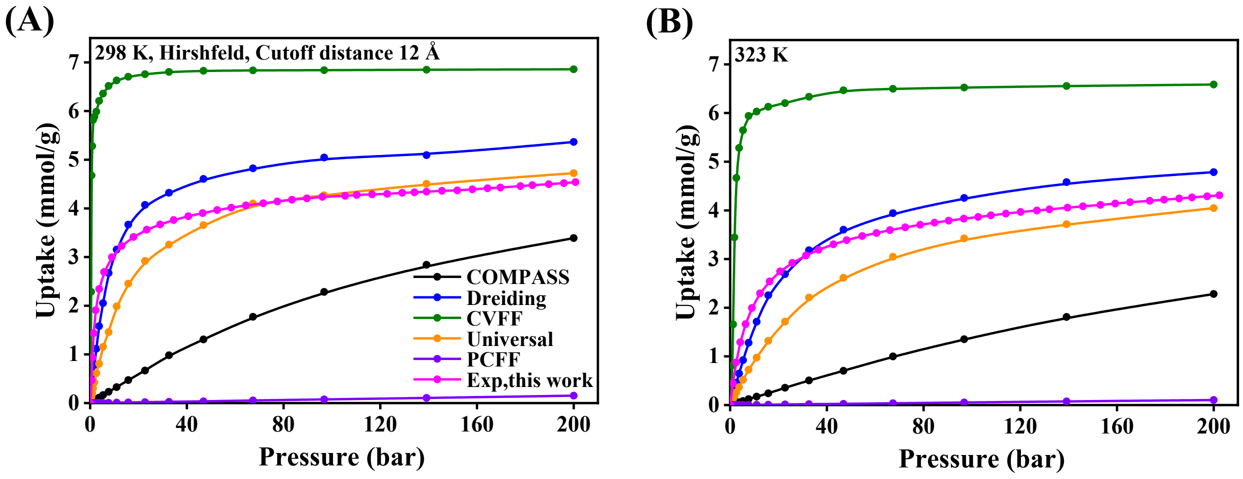

Figure. S3. Isotherms of CO2 adsorption in zeolite 5A with different force fields and a cut-off distance of 12 Å at (A) 298 K and (B) 323 K. 323 K.

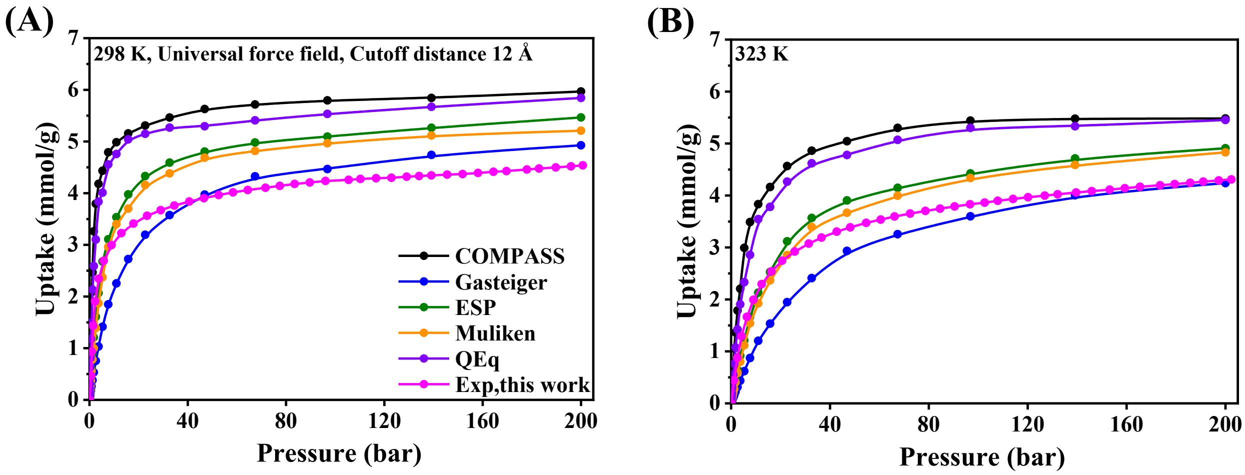

Figure S4. Isotherms of CO_2 adsorption in zeolite 5A with different charges at (A) 298 K and (B) 323 K, where multiple charges sets were studied for CO_2, while the zeolite 5A structure employed charges derived from Hirshfeld population analysis.

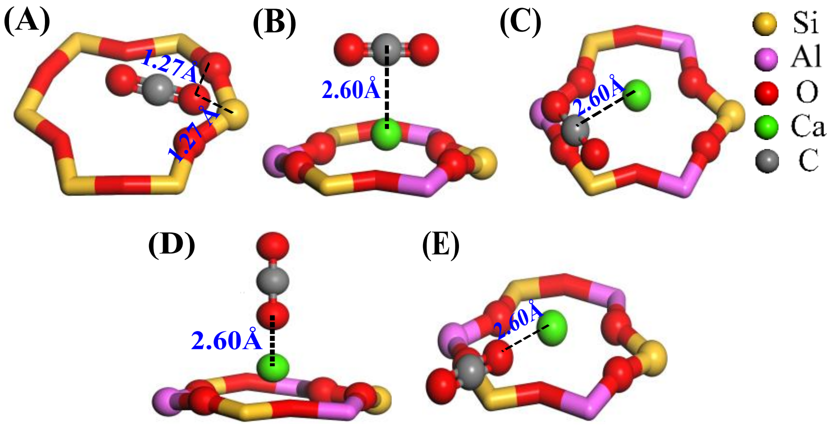

Figure S5. Initial configurations of CO_2 before geometry optimization on the surface of zeolite LTA. (A) Zeolite ITQ-29 with the CO_2 molecule positioned near O and Si atoms in a six-membered ring. (B, C) Horizontal configurations with the carbon atom of CO_2 placed near Ca^{2+} cation, and (D, E) Vertical configurations with the oxygen atom of CO_2 placed near Ca^{2+} cation.

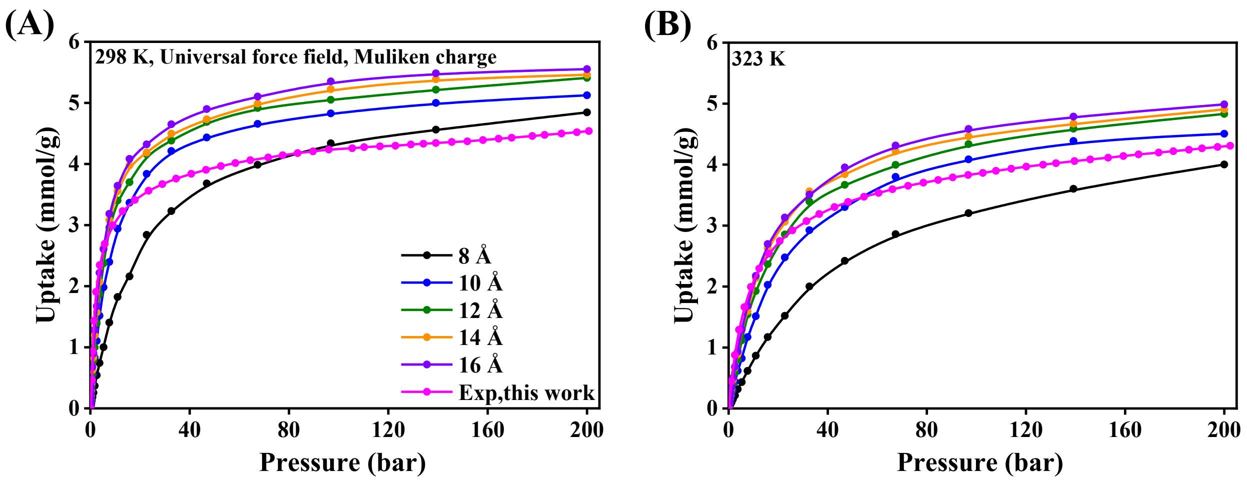

Figure S6. Isotherms of CO_2 adsorption in zeolite 5A with different cut of distance at (A) 298 K and (B) 323 K, where Muliken charge was applied for CO_2, and the zeolite 5A structure employed charges derived from Hirshfeld population analysis.

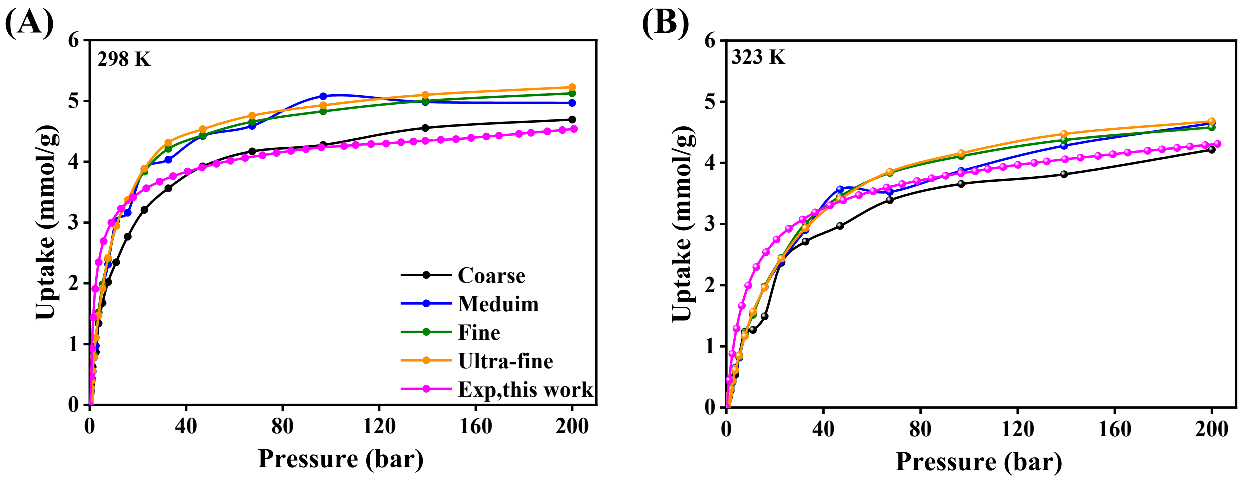

Figure S7. Convergence testing of equilibration and production steps at (A) 298 K and (B) 323 K, demonstrating the balance between computational accuracy and efficiency, with 10^5 steps for equilibrium and 10^6 steps for production (fine accuracy).

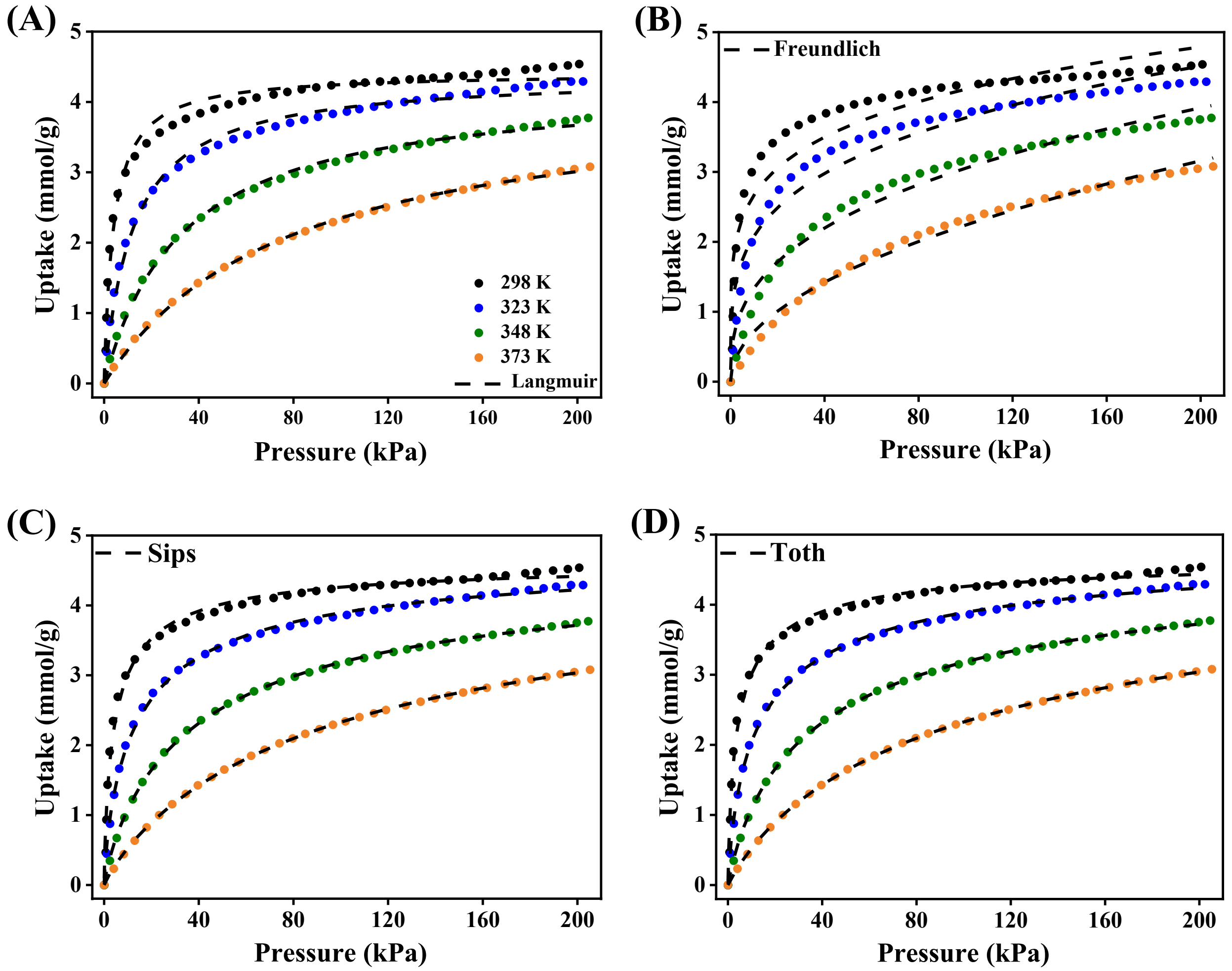

Figure S8. Adsorption isotherms of CO_2 in zeolite 5A at varying temperatures, fitted with (A) Langmuir, (B) Freundlich, (C) Sips, and (D) Toth models. The data demonstrates the adsorption behavior and model applicability, showing the relationship between the amounts of CO_2 adsorbed and the equilibrium pressure at different temperatures. Each model is evaluated to determine the best fit for the experimental data, providing insights into the adsorption capacity, surface heterogeneity, and the nature of CO_2-zeolite 5A interactions under varying thermodynamic conditions.

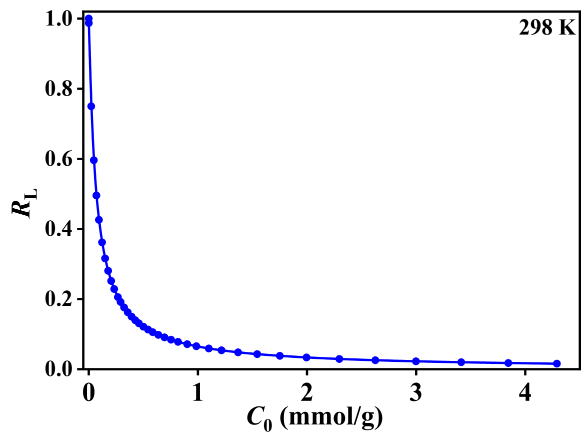

Figure S9. Separation factor R_L for CO_2 adsorption in zeolite 5A is < 1, indicating favorable adsorption.

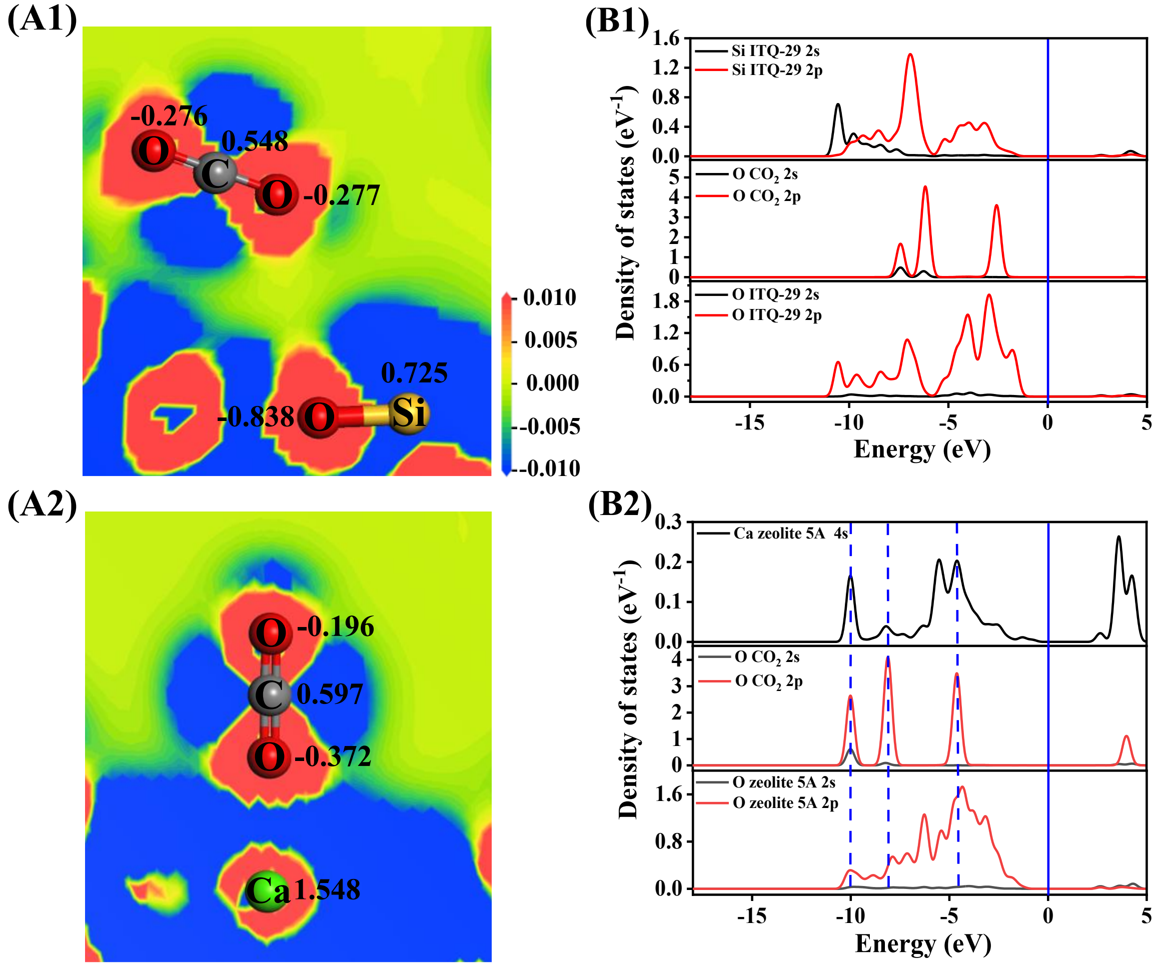

Figure S10. (A1, A2) Deformation charge density maps and (B1, B2) partial density of states (PDOS) for CO2 adsorption in zeolites ITQ-29 and 5A. A1 and B1 correspond to zeolite ITQ-29, while A2 and B2 correspond to zeolite 5A. The region colors of red and blue indicate electron enrichment and depletion region, respectively, and the numbers near the atoms are Mulliken charges. The Fermi level is set to 0 eV in the PDOS analysis.

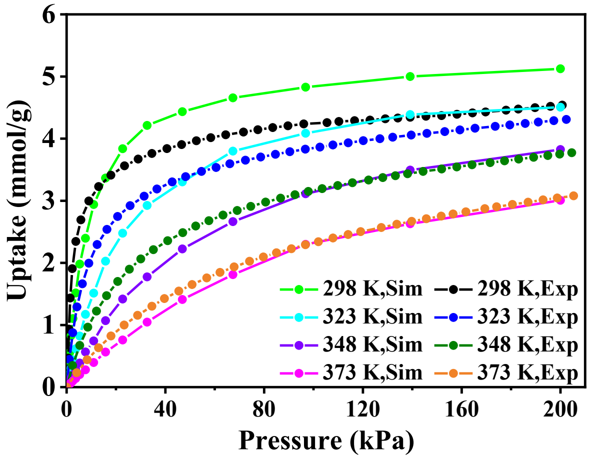

Figure S11. Comparison of simulation and experimental CO_2 adsorption data in zeolite 5A. The figure presents a detailed analysis of the adsorption isotherms, indicating the consistency and discrepancies between the simulation results and experimental observations. The comparison includes a range of temperatures and pressures, assessing the accuracy of the simulation model in predicting the adsorption behavior of CO_2 in zeolite 5A. This comparison provides insight into predictive capability of the model and its ability to replicate the real-world adsorption process.

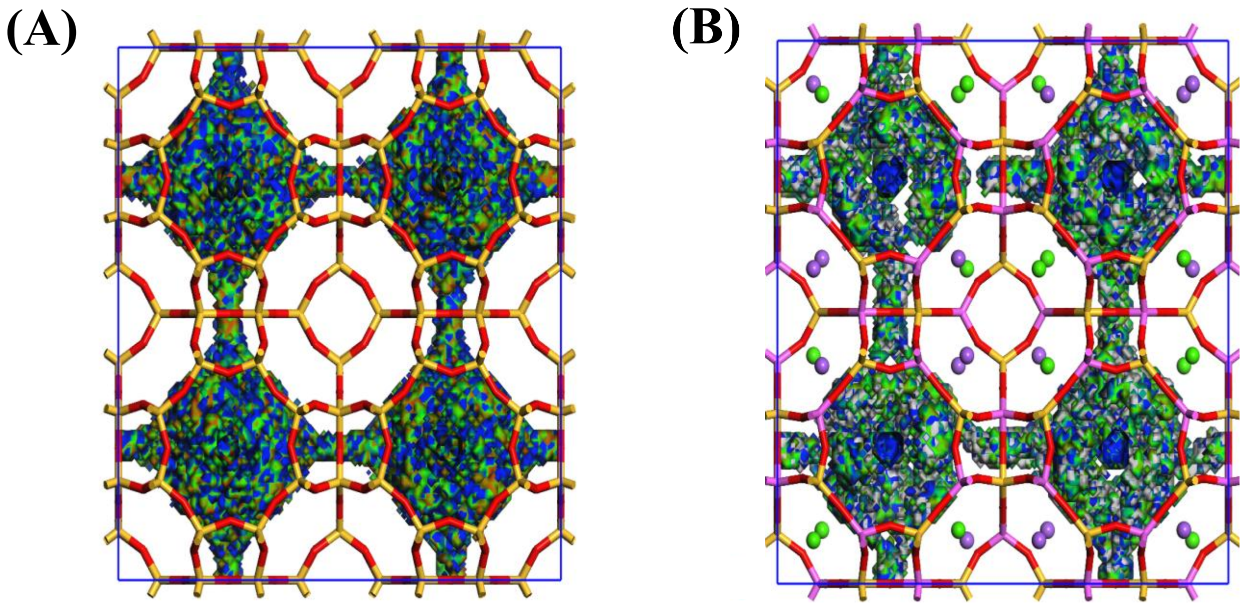

Figure S12. Isosurface energy fields of CO_2 adsorption in zeolites ITQ-29 and 5A. (A) Zeolite ITQ-29; broad range of interaction strengths (blue: strong, green: moderate, red: weak). (B) Zeolite 5A; only strong to moderate interactions (blue and green) due to framework cations. Sticks: yellow (Si), red (O), and pink (Al). Spheres: purple (Na), and green (Ca), gray (C).

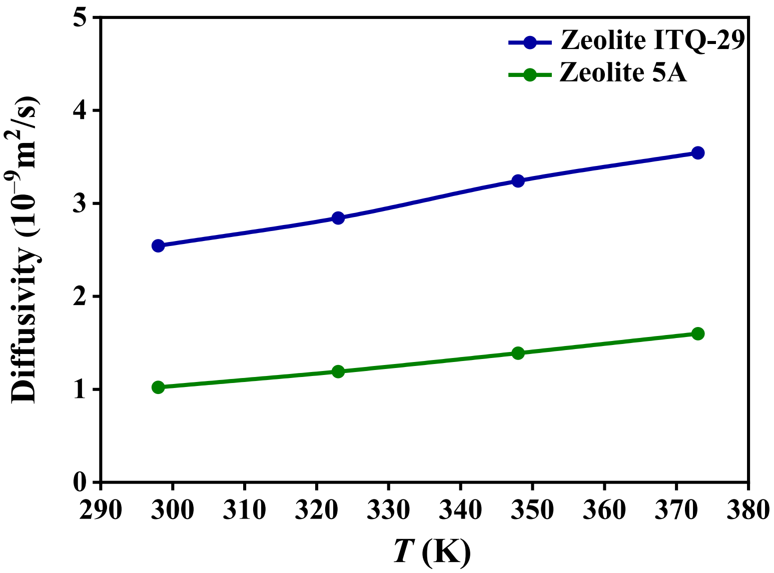

Figure S13. Diffusion coefficients as a function of temperature.

Table S1 Atomic coordinates of zeolite ITQ-29.

Atom	Before optimization [50]			After optimization		
	x	*y*	*z*	*x*	*y*	*z*
Si	0	0.370	0.184	0	0.364	0.182
O (1)	0	0.500	0.223	0	0.500	0.215
O (2)	0	0.291	0.291	0	0.306	0.306
O (3)	0.109	0.109	0.348	0.113	0.113	0.327

Table S2 Atomic coordinates of zeolite 5A.

Atom	Before optimization [51]			After optimization		
	x	*y*	*z*	*x*	*y*	*z*
Si	0	0.187	0.091	0	0.184	0.088
Al	0	0.091	0.187	0	0.088	0.184
O (1)	0	0.250	0.107	0	0.250	0.110
O (2)	0	0.140	0.140	0	0..143	0..143
O (3)	0.056	0.056	0.169	0.053	0.053	0.172
Ca	0.098	0.098	0.098	0.103	0.103	0.103
Na	0.080	0.080	0.080	0.077	0.077	0.077

Table S3 Average atomic charges derived from DFT calculations for CO_2 molecules and LTA zeolites used in the simulations.

Species	Atom	Charge
Zeolite ITQ-29	Si	0.510
	O	–0.255
Zeolite 5A	Si	0.479
	Al	0.403
	O	–0.307
	Na	0.397
	Ca	0.639
CO_2	C	0.544
	O	–0.272

Table S4 Adsorption parameters of the Toth model for CO_2 adsorption in zeolite 5A.

Temperature (K)	Adj. R^2	ΔQ	Model parameters		
			q_m (mmol/g)	k (kPa^{-1})	m
298	0.9935	8.90×10^{-3}	5.1887	0.4568	0.6489
323	0.9988	1.50×10^{-3}	4.9945	0.1443	0.6725
348	0.9998	2.55×10^{-4}	4.9559	0.0387	0.7204
373	0.9999	4.44×10^{-5}	4.7521	0.0132	0.7418

Table S5 Thermodynamic parameters of CO_2 adsorption in zeolite 5A.

Temperature (K)	ΔG^0 (kJ/mol)	ΔH^0 (kJ/mol)	ΔS^0 (J/mol·K)
298	–9.68	–44.04	–115.23
323	–6.80		
348	–3.92		
373	–1.03		

Table S6 Adsorption energies of CO_2 on different cation-exchanged sites.

Cation	Adsorption Energy (kJ/mol)
Na^+	–25.25
Li^+	–29.85
K^+	–21.57
Ca^{2+}	–47.13
Mg^{2+}	–63.77
